# The Effects of *Bifidobacterium* Probiotic Supplementation on Blood Glucose: A Systematic Review and Meta-Analysis of Animal Models and Clinical Evidence

**DOI:** 10.1016/j.advnut.2023.10.009

**Published:** 2023-11-02

**Authors:** Emily P. Van Syoc, Janhavi Damani, Zachary DiMattia, Erika Ganda, Connie J. Rogers

**Affiliations:** 1 Dual-Title Ph.D Program in Integrative & Biomedical Physiology and Clinical & Translational Science, The Pennsylvania State University, University Park, PA, United States; 2 Department of Animal Science, The Pennsylvania State University, University Park, PA, United States; 3 The One Health Microbiome Center, The Pennsylvania State University, University Park, PA, United States; 4 The Intercollege Graduate Degree Program in Integrative and Biomedical Physiology, Huck Institutes of Life Sciences, The Pennsylvania State University, University Park, PA, United States; 5 Department of Nutritional Sciences, The Pennsylvania State University, University Park, PA, United States; 6 Department of Nutritional Sciences, University of Georgia, Athens, GA, United States

**Keywords:** probiotics, *Bifidobacterium*, blood glucose, metabolic syndrome, type 2 diabetes mellitus, hemoglobin A1c

## Abstract

Probiotic supplementation is a potential therapeutic for metabolic diseases, including obesity, metabolic syndrome (MetS), and type 2 diabetes (T2D), but most studies deliver multiple species of bacteria in addition to prebiotics or oral pharmaceuticals. This may contribute to conflicting evidence in existing meta-analyses of probiotics in these populations and warrants a systematic review of the literature to assess the contribution of a single probiotic genus to better understand the contribution of individual probiotics to modulate blood glucose. We conducted a systematic review and meta-analysis of animal studies and human randomized controlled trials (RCTs) to assess the effects of *Bifidobacterium* (*BF*) probiotic supplementation on markers of glycemia. In a meta-analysis of 6 RCTs, *BF* supplementation had no effect on fasting blood glucose {FBG; mean difference [MD] = −1.99 mg/dL [95% confidence interval (CI): −4.84, 0.86], *P* = 0.13}, and there were no subgroup differences between subjects with elevated FBG concentrations and normoglycemia. However, *BF* supplementation reduced FBG concentrations in a meta-analysis comprised of studies utilizing animal models of obesity, MetS, or T2D [*n =* 16; MD = −36.11 mg/dL (CI: −49.04, −23.18), *P* < 0.0001]. Translational gaps from animal to human trials include paucity of research in female animals, *BF* supplementation in subjects that were normoglycemic, and lack of methodologic reporting regarding probiotic viability and stability. More research is necessary to assess the effects of *BF* supplementation in human subjects with elevated FBG concentrations. Overall, there was consistent evidence of the efficacy of *BF* probiotics to reduce elevated FBG concentrations in animal models but not clinical trials, suggesting that *BF* alone may have minimal effects on glycemic control, may be more effective when combined with multiple probiotic species, or may be more effective in conditions of hyperglycemia rather than elevated FBG concentrations.

**Statement of Significance**

To our knowledge, this is the first systematic review and meta-analysis on the effects of one probiotic genus, *Bifidobacterium*, on blood glucose that does not include multigenus probiotic mixtures or the additional use of prebiotics or antidiabetic therapy. The review additionally discusses findings from animal studies to further understand how *Bifidobacterium* supplementation may affect blood glucose in conditions of obesity, metabolic syndrome, and type 2 diabetes.

## Introduction

There is growing interest in the use of probiotic supplementation to ameliorate metabolic diseases including obesity, metabolic syndrome (MetS), and type 2 diabetes (T2D). However, variability in the genera and species used and inconsistencies in the duration, dose, and delivery methods of probiotic supplementation have led to conflicting results and hindered our understanding of the efficacy of probiotic supplementation in these populations [[1], [2], [3]]. To address this variability and further understand the contribution of individual probiotic genera, the current review specifically focuses on the use of *Bifidobacterium* (*BF*) species to modulate blood glucose in subjects with obesity, MetS, or T2D. MetS is characterized by the presence of central obesity in addition to 2 or more additional factors including dyslipidemia, reduced HDL cholesterol, hypertension, or elevated fasting plasma glucose (FBG; >100 mg/dL) concentrations [4]. T2D is defined as sustained hyperglycemia, which is considered as FBG ≥126 mg/dL or hemoglobin A1c (HbA1c) ≥6.5% [5]. Elevated FBG concentration is an independent risk factor for cardiovascular disease, and people with MetS are a significant risk of developing T2D among other chronic diseases [[6], [7], [8]]. Thus, adults with obesity and elevated FBG concentrations and/or additional MetS criteria are a target population of preventative therapies to mitigate T2D risk.

Probiotics have been evaluated as a potential therapeutic for glycemic modulation in the conditions of elevated FBG concentrations, MetS, and T2D [9]. The prevalence of metabolic diseases (obesity, elevated FBG concentrations, MetS, or T2D) is escalating, and probiotic supplementation may present a low-cost and low-risk intervention to improve metabolic health [10,11]. This warrants a systematic review of the existing literature to determine the extent to which probiotic supplementation modulates blood glucose concentrations in metabolic disease, as existing reviews have yielded conflicting results [[1], [2], [3],[12], [13], [14], [15]]. A recent study of 47 meta-analyses found an overall favorable effect of probiotic and/or prebiotic supplementation on FBG in a heterogeneous population of subjects with obesity, MetS, or T2D among additional disorders [9]. These and other meta-analyses of probiotic supplementation have included the use of any single probiotic bacteria and/or a combination of bacterial genera or yeasts, and most included the additional use of prebiotics and/or antidiabetic therapy [[1], [2], [3],[12], [13], [14], [15]]. Strain-specific effects, interactions with prebiotics, and mixtures of different species could differentially affect blood glucose regulation and the overall efficacy of probiotic supplementation. Indeed, genomic comparisons of *Lactobacillus* and *BF* demonstrate large gene families and functional differences between these genera and even between strains from the same species [16,17]. In addition, reviews, including animal studies demonstrating an effect of probiotic supplementation in animal models with pathophysiology relevant to MetS or T2D are lacking. Thus, there is a pressing need for a focused, narrow review on one type of probiotic bacteria without the confounding factors of multiple probiotic genera, prebiotics, or pharmaceuticals on measures of glycemic control to determine if potential benefit may be achieved.

*BF* is one of the most commonly used lactic acid-forming probiotic genera [18]. Health benefits conferred by *BF* supplementation may include upregulating autophagy signaling in goblet cells and improving intestinal mucus layers [19], adhering to enterocytes to competitively exclude pathogens [20], increasing folic acid production [21], and fermenting larger polysaccharides to provide energy to other microbes [22]. *BF* species are attractive probiotics because they are culturable, survive transit through the upper gastrointestinal tract, and are historically considered safe for human consumption [23]. To date, to our knowledge, no systematic reviews of the existing literature have examined the effect of *BF* as the sole probiotic genus on glycemic control in humans or animal models with elevated FBG concentrations. Thus, we conducted a systematic review and meta-analysis of human and animal studies to determine if *BF* probiotic supplementation modulates glycemic markers in animal models of obesity, MetS, or T2D and/or populations with these metabolic disorders. To further understand the extent to which *BF* may modulate blood glucose, the analysis of animal experiments was subset to address the following 3 distinct research questions: *1)* does *BF* affect blood glucose concentrations in healthy animals; *2)* does *BF* lower blood glucose concentrations in models of obesity/MetS/T2D, and *3)* does *BF* lower blood glucose concentrations in models of obesity/MetS/T2D to a concentration comparable with healthy, untreated animals. Similarly, a subgroup analysis was performed of clinical trials [randomized controlled trial (RCTs)] to determine if *BF* differentially affected blood glucose concentrations in adult populations with normoglycemia or elevated FBG concentrations (>100 mg/dL). We additionally sought to identify discrepancies between animal studies and RCTs that could result in translational gaps.

## Methods

### Protocol registration and search strategy

This systematic review was conducted using the PRISMA statement [24] and was prospectively registered in PROSPERO (CRD42022384180). A search strategy was developed in collaboration with a health science librarian at the Pennsylvania State University and included 2 groups of terms, including probiotic supplementation with *BF* (exposure) and glycemic control (outcome) in both humans and animal models (population) to evaluate the evidence linking dietary supplementation with one or more species of *BF* and indicators of glycemic response [FBG, HbA1c, or oral/intraperitoneal glucose tolerance test (OGTT/IPGTT)]. A systematic literature search was conducted using PubMed (Medline), Web of Science, and CAB Direct on 8 December 2022. The full search terms are described in the supplemental material (Supplemental Table 1).

### Inclusion and exclusion criteria

Only primary research articles written in the English language were included in this review. Inclusion criteria for both preclinical and clinical studies included a probiotic intervention arm and ≥1 measure of glycemic control. Human populations included adult subjects (>18 y old) of any sex, race, or population. Animal populations were categorized into models of obesity, MetS, or T2D. Diet-induced obesity (DIO) or transgenic (TrG) obesity models were classified separately from MetS models to differentiate obesity from the cluster of additional criteria that define MetS [25]. To be classified as a model of MetS, the animal model needed to establish obesity in addition to ≥1 MetS criteria, such as dyslipidemia, hypertension, or hyperglycemia (e.g., obese Zucker rats reliably establish hyperlipidemia and insulin resistance) [25,26]. T2D animal models included diet-induced T2D, TrG strains, or chemically induced T2D with streptozotocin (STZ). STZ injections selectively damage islet β cells and impair insulin secretion [27]. To induce a T2D phenotype, a high-fat diet (HFD) is used to impair glucose regulation and insulin secretion is further impaired by a moderate STZ dose [27]. Animals were considered healthy if they were normoglycemic, nonobese, and on a standard (nonhigh fat) diet. Studies that used additional interventions, such as prebiotics, in conjunction with probiotics, or mixtures of multiple probiotic species were excluded. Further inclusion and exclusion criteria are described in Supplemental Table 2.

### Study selection, data extraction, and analysis

Complete searches of all 3 databases were conducted to obtain references for inclusion. A total of 1150 references were exported to Mendeley version 2.81.0 (Supplemental Figure 1). After de-duplication, 618 references underwent title and abstract screening by 2 independent investigators to identify potentially eligible studies and conflicts were resolved with a third reviewer. The full texts of the identified studies were investigated independently with reference to the inclusion and exclusion criteria. Data were extracted into a standardized spreadsheet and included bibliographic information (author and publication year), country of origin, animal model or human subject characteristics, reported probiotic strain and mode of delivery, probiotic dosage, length of intervention, and markers of glycemic control including FBG, HbA1c, 2-h OGTT/IPGTT, and OGTT/IPGTT AUC, fructosamine, or glycated albumin.

### Quality assessment

For all animal studies, quality was assessed using the Systematic Review Centre for Laboratory Animal Experimentation’s Risk of Bias tool, which is based on the Cochrane Risk of Bias (RoB) tool [28]. Using this tool, quality is determined on 10 questions for the following 6 types of bias; selection bias, performance bias, detection bias, attrition bias, reporting bias, and other biases (Supplemental Table 3). In this table, a higher quantity of “yes” or “probably yes” answers are indicative of higher quality studies. For all clinical interventions, quality was assessed using the second version of the Cochrane RoB tool [29]. Quality was assessed based on entries for the following 5 domains of bias: randomization process, deviations from the intended interventions, missing outcome data, measurement of the outcome, and reported result (Supplemental Table 4). Using the flowchart guidance from Cochrane, each domain was assigned a risk of bias as “low,” “some concerns,” or “high.” This paper followed the PRISMA guidelines (Appendix A).

### Statistical analyses of meta-analysis

Meta-analysis was performed with the “meta,” “dmetar,” and “metafor” packages in R version 4.2.1 [[30], [31], [32]]. All results are presented as mean [95% confidence interval (CI)]. Statistical significance was accepted at *P* value of <0.05. The mean and SD of each glycemic variable were pooled into mean differences (MDs) and 95% CI using a Knapp–Hartung adjustment [33]. Cohen’s *d* effect size is additionally reported for generalizability [34]. Studies that tested multiple probiotic species in independent groups were included separately in the analysis. Where necessary, SE was converted to a standard deviation with the equation $sd=SEM\times \sqrt{n}$ [35]. Probiotic supplementation duration was converted from days to weeks. Blood glucose concentrations and 2-h OGTT were converted from mmol/L to mg/dL with the equation $mg{dL}^{-1}=18\times mmolL^{-1}$ (https://www.diabetes.co.uk/blood-sugar-converter.html). HbA1c was converted from mmol/mol to percentage (%) with the equation $\%=0.0915\;mmol{mol}^{-1}+2.15$ (https://www.diabetessociety.com.au/documents/HbA1cConversionTable.pdf). Meta-analysis was conducted with a random effects model and visualized with forest plots. Heterogeneity was assessed with the *I*^*2*^ statistic, and *I*^*2*^ >50% was considered moderate [36,37]. A leave-one-out approach, implemented with the “meta” R package, was used to detect potentially influential studies, and sensitivity to the identified influential studies was assessed by running the random effects model with and without the influential studies. In animal studies, 3 separate analyses were conducted. These include the following: *1*) *BF-*supplemented healthy compared with untreated healthy animals, *2*) *BF-*supplemented models of metabolic disease (obese/MetS/T2D) compared with untreated metabolic disease animals, and *3*) *BF-*supplemented models of metabolic disease (obese/MetS/T2D) compared with untreated healthy animals. In RCTs, a subgroup analysis was performed with a Chi-squared test, as implemented in the “meta” R package, to assess differences in elevated FBG concentrations (baseline mean FBG > 100 mg/dL) compared with subjects with normoglycemia [6]. Subgroup analysis was not possible in the meta-analysis of HbA1c because fewer studies reported HbA1c. To assess publication bias, funnel plots were created, and bias was quantitatively confirmed with Egger’s regression test when the sample size was sufficient [38].

## Results

### Quality assessments

Quality assessments revealed a moderate to high risk of bias in some of the animal studies (*n =* 19) and RCTs (*n =* 4). The methodologic descriptions of several preclinical studies were brief and did not elaborate sufficiently to determine whether a risk of bias was present or not (Supplemental Table 3). This problem was not isolated to animal experiments because 4 of the RCTs were characterized by a high risk of bias (Supplemental Table 4).

### Review of animal studies

#### Search summary.

A total of 40 animal studies utilizing models with pathophysiology relevant to obesity, MetS, or T2D met inclusion criteria and were included in this review (Supplemental Figure 1). The most common DIO models were HFD-fed C57Bl/6 mice and Sprague–Dawley rats; obese Zucker rats were frequently used as a TrG MetS model; and STZ injections were used more than TrG strains to model T2D. Studies that tested multiple experimental designs or multiple probiotic strains are reported independently and are differentiated with superscript letters after the study identifier. The most commonly reported *BF* species were *B. animalis* (*n =* 19), *B. bifidum* (*n =* 18), *B. longum* (*n =* 16), and *B. adolescentis* (*n =* 14). Less common species were *B. breve* (*n =* 5), and *B. pseudocatenulatum* (*n =* 2). Three studies used a mixture of multiple *BF* species, and 5 studies did not report the *BF* species. Probiotics were mostly delivered via daily oral gavage, although a few studies added probiotics to feed pellets or drinking water. Dosage and duration varied widely. A complete description of experimental designs, animal models, and glycemic results are reported in Supplemental Table 5.

#### BF supplementation in healthy animals.

Indicators of glycemic control (FBG, HbA1c, and OGTT/IPGTT) were compared following *BF* supplementation in healthy (nonobese and nondiabetic), standard feed pellets-fed animals compared with healthy control animals with no *BF* supplementation. One study noted increased FBG concentrations in Wistar rats with 10^10^ colony forming units (CFU) of heat-killed *B. animalis* spp. *lactis* CECT-8145 supplemented in feed pellets for 12 wk (175.68 ± 14.04 mg/dL) compared with the standard-fed counterparts without probiotic supplementation (152.46 ± 18.9 mg/dL) [39]. This is the only study that reported increased FBG concentrations following probiotic supplementation compared with untreated, healthy rats. All other studies reported no differences in FBG (*n =* 6; Salazar 2014 reported on 2 different *BF* species) between *BF-*supplemented healthy animals and untreated healthy animals [[40], [41], [42], [43], [44], [45]] and/or OGTT-AUC/IPGTT AUC (*n =* 3) [40,46,47]. These findings were consistent despite variability in animal phenotypes, probiotic species, dose, delivery methods, and duration (Supplemental Table 5A). All animal studies were conducted in male animals, except one that used female Balb/c mice [45]. There was general agreement that *BF* did not affect blood glucose in healthy animals.

#### BF supplementation in models of metabolic disease.

To determine if *BF supplementation* modulates markers of glycemic control in animal models of obesity, MetS or T2D, glycemic markers (FBG, HbA1c, and OGTT/IPGTT) in *BF-*supplemented animals were compared with control, untreated animal models of obesity, MetS, or T2D. In 22 studies, HFD was used to induce obesity. In 10 studies that used DIO C57Bl/6 mice, *BF* supplementation for 6–16 wk decreased FBG concentrations or OGTT/IPGTT compared with untreated controls [40,42,[47], [48], [49], [50], [51], [52], [53], [54], [55]]. This included studies that tested >1 strain or dose. Two studies found no differences in FBG or OGTT/IPGTT between probiotic-supplemented and nonprobiotic-supplemented control groups [56,57], whereas 1 study reported decreased OGTT-AUC after 7 wk of probiotic supplementation with 2 strains of *B. animalis* but no differences in supplementation with *B. longum* [48]. Rodent models of DIO using Sprague–Dawley rats, Wistar rats, or other rodent species also reported that *BF* supplementation for 4–8 wk decreased FBG concentrations or OGTT/IPGTT (*n =* 5) [46,[58], [59], [60], [61]]. However, 4 studies found no differences in probiotic and non-supplemented groups, including 1 study that tested 4 different *BF* strains [49,[62], [63], [64]]. One study that evaluated 7 different *BF* strains in HFD-fed Sprague–Dawley rats found conflicting results. Rats that were individually administered with a single strain of *B. longum*, *B. adolescentis*, or *B. bifidum* for 12 wk exhibited decreased FBG concentrations and OGTT-AUC compared with untreated controls. However, some strains of *B. breve* and *B. bifidum* showed contradictory effects, with *B. breve* “R2” and *B. bifidum* “F35” exerting no effect on FBG or OGTT, and *B. breve* “S13” increased OGTT-AUC. Further, 1 strain of *B. longum* decreased FBG concentrations with no effect on OGTT-AUC strain “R2” exerting no effect on OGTT-AUC whereas the other “S13” strain increased OGTT-AUC (Supplemental Table 5B) [65]. Animal studies utilizing models of DIO in C57Bl/6 mice reported consistent findings of decreased FBG concentrations or OGTT/IPGTT after *BF* supplementation, whereas findings reported from studies conducted in other DIO models were highly variable.

Obese Zucker rats were used in 3 studies. This strain is an established model of MetS that develops obesity, dyslipidemia, insulin resistance, and hyperglycemia among additional metabolic aberrations, regardless of diet [26]. All 3 studies found no differences in FBG concentrations between *BF-*supplemented obese Zucker rats and untreated rats, with supplementation duration ranging from 4 to 14 wk [[66], [67], [68]]. Two studies generated DIO models of MetS using HFD or in 1 case, a “cafeteria diet” comprised of bacon and additional foods added to standard feed pellets. Both studies found no difference in FBG concentrations after 12 wk of *BF* supplementation compared with untreated MetS animals [39,69]. *BF* supplementation did not modulate blood glucose in TrG (all obese Zucker rats) or DIO models of MetS.

Pathophysiology relevant to T2D was chemically induced in 4 studies with STZ injections after HFD feeding (abbreviated as DIO-STZ-D). Two studies found decreased FBG concentrations and OGTT-AUC after 6–10 wk of *BF* supplementation in mice and rats [70,71]. Two studies tested multiple strains, administered separately (not as a mixture), with varied results. Qian et al. [72] supplemented 1 of the 16 *BF* strains for 7 wk in mice alongside HFD feeding, then administered STZ and continued probiotic supplementation and HFD for an additional 5 wk. Supplementation of 3 strains of *B. adolescentis* resulted in decreased OGTT-AUC compared with untreated diabetic mice, whereas an additional 5 strains of *B. adolescentis* and 8 strains of *B. bifidum* did not affect OGTT-AUC [72]. The second study supplemented probiotics for 5 wk after STZ administration in mice and found that 2 strains of *B. adolescentis* and one strain of *B. bifidum* lowered FBG concentrations, OGTT, and HbA1c concentrations whereas another strain of *B. bifidum* did not affect any glycemic metric, and a strain of *B. adolescentis* decreased OGTT-AUC but did not affect FBG or HbA1c concentrations [73]. TrG models of T2D (*n =* 7) had varied findings. In 5 studies, *BF* supplementation ranging from 2 to 13 wk decreased FBG concentrations or OGTT/IPGTT [48,54,[74], [75], [76]]. This included one study that reported decreased FBG concentrations and 2-h OGTT with supplementation of autoclaved *B. longum* in mice at doses of 100 and 150 mg/kg but no effects at a 50 mg/kg dose [74]. One study used 2 different TrG strains and reported decreased FBG concentrations after 2 wk of *B. breve* supplementation in KK-A^y^ mice but no differences in FBG concentrations after 3 wk of *B. breve* supplementation in obese Wistar rats [76]. In 1 study, DIO induction of T2D using feed pellets containing 72% fat, and *BF* supplementation for 6 wk had no effect on FBG concentrations or IPGTT/OGTT [53]. In both STZ and TrG models of T2D, *BF* supplementation generally decreased glycemic metrics, but with some conflicting results. This suggests that animal models, diabetic severity, and strain-specific effects may affect the efficacy of *BF* to modulate FBG in models of T2D.

The most commonly reported *BF* species was *B. animalis* (*n =* 19) with 18 reported as the subspecies *B. animalis* spp. *lactis*. This strain was generally effective at reducing glycemic metrics when supplemented with animal models of obesity, MetS, or T2D. Twelve of the studies using *B. animalis* found decreased FBG concentrations, OGTT, or HbA1c concentrations compared with untreated obese, MetS, or T2D control animals, whereas 6 studies found no differences (Supplemental Table 5). Supplementation duration ranged from 2 to 12 wk in the studies that found no differences. Similarly, most of the studies (*n =* 8 and 11, respectively) that reported *B. longum* or *B. bifidum* found a decrease in ≥1 glycemic metric whereas fewer observed no differences (*n =* 3 and 2, respectively). *B. breve* was less frequently reported, but only 1 study reported its efficacy in decreasing FBG concentrations [51] whereas 2 studies, 1 of which tested 2 different strains of *B. breve*, reported no differences [65,68]. Overall, the commonly reported *BF* species lowered FBG concentrations or other glycemic metrics in animal models of obesity, MetS, or T2D compared with untreated control animals regardless of treatment duration or dose.

#### BF supplementation in models of metabolic disease compared with healthy animals.

To determine if *BF* supplementation reduced elevated FBG concentrations to a level that is statistically similar to healthy animals, studies of *BF* supplementation in models of obesity, MetS, or T2D were compared with untreated, healthy controls. Nine studies, some of which tested multiple strains, used an HFD to induce obesity in C57Bl/6 mice. Six studies found no differences in FBG concentrations or OGTT/IPGTT between *BF-*supplemented obese mice and untreated healthy mice [40,42,47,50,52,56], whereas 4 studies found that *BF-*supplemented mice retained higher FBG concentrations or OGTT/IPGTT compared with healthy controls [48,52,53,55]. In these studies, probiotics were supplemented for 6–13 wk. DIO in Wistar rats, Sprague–Dawley rats, Swiss mice, or Albino mice (*n =* 5) generally agreed that *BF* supplementation for 2–12 wk reduced FBG concentrations or OGTT/IPGTT in obese animals to similar levels as healthy, untreated controls, including 1 study that tested 4 different strains [46,58,61,63,64]. One study that supplemented irradiated *B. animalis* spp. *lactis* BB-12 in pasta found decreased FBG concentrations in the probiotic group compared with untreated, healthy rats on standard feed pellets without pasta [62]. Another study tested 7 different *BF* strains and found strain-specific results; 1 strain of *B. breve* “S13” and 1 strain of *B. bifidum* “F35” resulted in higher FBG concentrations and OGTT-AUC in DIO Sprague–Dawley rats compared with healthy control rats, whereas 2 strains of *B. longum* “C-1 A4” and “K2” resulted in similar FBG, but higher OGTT-AUC compared with healthy rats. In contrast, 1 strain of *B. breve* “R2” had similar OGTT-AUC but increased FBG concentrations compared with healthy rats, and 1 strain of *B. adolescentis* “Z25” had similar FBG and OGTT-AUC compared with healthy rats (Supplemental Table 5B) [65]. Apart from the heterogeneity present in those few studies, *BF* supplementation reduced blood glucose in animal models of DIO to a level comparable to healthy animals.

In 1 study utilizing a model of MetS with obese Zucker rats, *B. animalis* spp. *lactis* strain CECT8145 supplementation for 12 wk at 10e^10^ CFU in drinking water resulted in increased FBG concentrations compared with lean Zucker rats, or rats without the mutation that causes metabolic disease [66]. A second study that delivered *B. breve* CNCM I-4035 to obese Zucker rats at 10^10^ CFU in oral gavage resulted in similar FBG to lean Zucker rats [68]. In models of MetS generated with DIO, there was no difference in FBG between MetS and healthy animals after supplementing the MetS rats with *B. longum* for 12 wk [69]. More research is needed to conclude the efficacy of *BF* to modulate FBG concentrations in animal models of MetS.

In models of T2D induced with chemical injections (DIO-STZ-D), 2 studies reported that *BF* supplementation in DIO-STZ-D had higher FBG concentrations compared with the untreated, nondiabetic control mices [72,77]. This included 1 study that tested 16 different *BF* strains and gave STZ injections after 7 wk of initial probiotic supplementation as a preventative therapy [72]. Similarly, 1 study found higher FBG concentrations, OGTT-AUC, and HbA1c in the DIO-STZ-D group compared with healthy control mice in 6 of the 7 *BF* strains [73], and 1 study found higher FBG concentrations and OGTT-AUC in the *BF-*supplemented DIO-STZ-D group compared with the nondiabetic, untreated control mice, but there was no difference in 2-h OGTT [70]. These studies highlight potential strain variability within the *BF* genus, although 2 studies obtained the different strains from a culture collection [72,73] and 1 isolated and cultured the strains in-house [70]. None reported verification of the probiotic composition or other differences in the integrity or viability of the strains that could explain the varied results (Supplemental Table 5). Three studies used TrG species to induce T2D pathophysiology, with varied results of *BF* supplementation on FBG or OGTT in the diabetic model compared with the healthy, untreated control animals. One study found similar FBG and 2-h OGTT in *BF-*supplemented T2D and healthy, nondiabetic control mice after 4.3 wk of supplementation [74]. In obese Wistar rats, FBG concentrations were higher in the *BF-*supplemented T2D compared with the lean Wistar rat controls after 3 wk of supplementation [76]. Finally, FBG and OGTT-AUC were decreased after 8 wk of *BF* supplementation compared with the healthy control mice [78]. Reports of FBG, OGTT/IPGTT, and HbA1c following *BF* supplementation in animal models of T2D varied, which may be partially because of heterogeneity in experimental models, disease severity, and probiotic supplementation duration and delivery methods. Overall, *BF* supplementation successfully modulated glycemic metrics in animals with pathophysiology relevant to T2D to similar levels as healthy controls in a few studies, but most reported that FBG concentrations remained higher than those in healthy, untreated controls.

There were no clear patterns that demonstrated a benefit of particular supplementation duration over others. For example, 1 study using irradiated *B. animalis* spp. *lactis* BB-12 observed decreased FBG concentrations compared with healthy controls after 2.1 wk of supplementation [62], whereas another using the BB-12 strain found no differences in OGTT-AUC after 10 wk of supplementation [52]. Similarly, 2 additional studies using strains of *B. animalis* found no difference in FBG concentrations [53,63] whereas 4 found increased FBG concentrations compared with healthy controls [48,52,53,66]. Various strains of *B. longum* were generally more successful as 8 of the studies found decreased FBG concentrations or no differences between supplemented and healthy control animals [46,47,50,65,69,74,78] whereas 3 found increased FBG concentrations [55,70,77]. Conversely, all but 1 study using strains of either *B. bifidum* (*n =* 13) or *B. adolescentis* (*n =* 8) found increased FBG concentrations compared with healthy animals, even with durations ranging from 3 to 12 wk. This included 3 studies that tested 2 [65], 3 [73], or 8 [72] different reported strains of *B. bifidum* and found that all supplementation of all strains resulted in increased FBG concentrations compared with healthy animals with the exception of one strain, “W25” [65]. Similarly, 8 different reported strains of *B. adolescentis* were tested in 1 study and all had higher OGTT-AUC compared with healthy animals [72], whereas a second study found no differences in FBG concentrations [56]. Thus, *B. longum* may be more effective in controlling FBG in models of DIO, MetS, or T2D compared with healthy animals without probiotic supplementation.

### Meta-analysis using data from animal studies

A total of 17 animal studies including 518 total animals were included in the meta-analysis. All 17 studies reported FBG, whereas only 4 reported HbA1c and 2 reported OGTT/IPGTT, so only FBG is reported in the meta-analysis. Studies that included multiple probiotic strains or experimental designs are reported independently and denoted with a letter following the study identifier.

### BF supplementation in healthy animals

**FIGURE 1:**
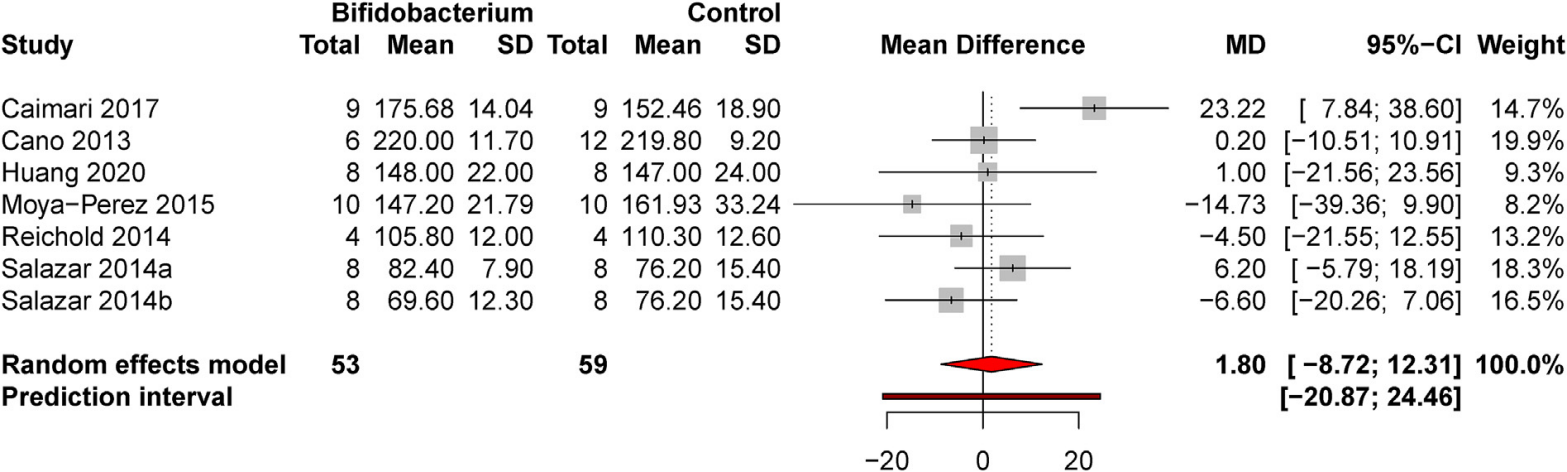
*Bifidobacterium* supplementation does not affect fasting blood glucose in healthy animals. A forest plot shows the pooled mean difference between healthy animals treated with probiotic supplementation compared with no supplement controls. No influential studies were removed from the effect size estimate.

Six studies including 1 study that tested 2 probiotic strains compared healthy animals with and without probiotic supplementation. No potentially influential studies were identified, and the pooled MD estimate was 1.80 mg/dL (8.72, 12.31) [*t* = 0.42, *P* = 0.69, and *d* = 0.09 (−054, 0.72)], suggesting no effect of *BF* supplementation on blood glucose in healthy animals when compared with similar, untreated healthy animals (Figure 1). Heterogeneity was moderate in this comparison [*I*^*2*^ = 48.9% (0.0, 78.4)] but publication bias was minimal (Supplemental Figure 2).

### BF supplementation in models of metabolic disease

**FIGURE 2:**
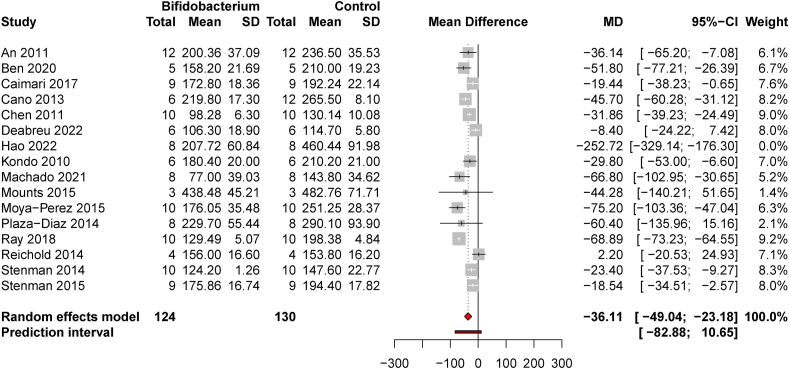
*Bifidobacterium* supplementation decreases fasting blood glucose in animal models with pathophysiology relevant to obesity, MetS, or T2D. A forest plot shows mean differences in 15 studies with models of DIO, MetS, or T2D with 1 potentially influential study (Hao et al. [70]) excluded from the effect size estimates.

A total of 16 studies and 254 animals were included in this comparison. Three of the studies used models of T2D whereas 13 used models of obesity or MetS. One influential study was removed which did not affect significance, but heterogeneity remained high [*I*^*2*^ = 92.6% (89.4, 94.8)]. The pooled estimate suggested a significant decrease in FBG concentrations following *BF* supplementation [MD = −36.11 mg/dL (−49.04, −23.18), *t* = −5.99, *P* < 0.0001, and *d* = −1.81 (−3.02, −0.61)] (Figure 2). Publication bias was minimal [Egger’s test intercept = 1.81 (−0.91, 4.54), *t* = 1.31, and *P* = 0.21; Supplemental Figure 3].

### BF supplementation in models of metabolic disease compared with healthy animals

**FIGURE 3:**
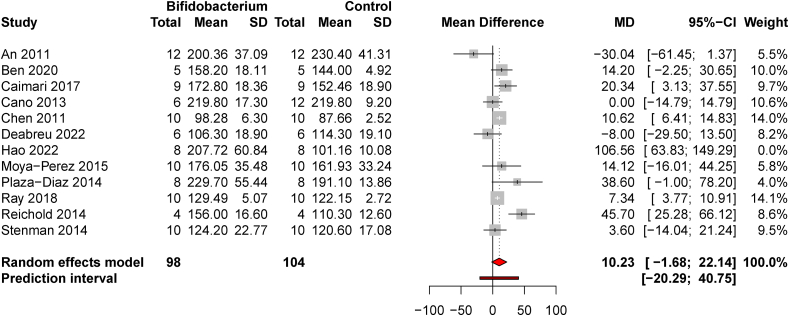
Fasting blood glucose in probiotic-supplemented animal models with pathophysiology relevant to obesity, MetS, or T2D compared with healthy, untreated normal-weight animals. A forest plot shows mean differences of 12 DIO, MetS, or T2D studies with 1 potentially influential study (Hao et al. [70]) removed from the effect estimates.

Twelve studies with 196 total animals were included in the comparison of *BF-*supplemented models of obesity, MetS, or T2D to untreated, healthy animals. Two studies used models of T2D whereas 10 used models of DIO or MetS. One influential study was removed from the effect size estimate, which did not affect significance. The pooled effect size was marginally nonsignificant [MD = 10.23 mg/dL (−1.68, 22.14), *t* = 1.91, *P* = 0.08, and *d* = 0.74 (0.02, 1.45)] (Figure 3). Heterogeneity was moderate [*I*^*2*^ = 64.6% (32.5, 81.4)]. Publication bias was minimal [Egger’s test intercept = 0.86 (−0.81, 2.53), *t* = 1.0, and *P* = 0.34; Supplemental Figure 4].

### Systematic review of clinical data

#### Search summary.

Eight human clinical trials met inclusion criteria and are included in this review, of which 6 are additionally included in the meta-analysis. The sample sizes ranged from 8 to 100 subjects per group and were all conducted in adult populations with primary inclusion criteria of obesity or MetS. The ratio of men to women varied widely and most were not stratified evenly by gender into probiotic and placebo arms (Table 1). Exclusion criteria for 4 studies included the use of most pertinent adjuvant, pre-existing medications, such as antidiabetic, antihypertensive, and statin medications [47,[79], [80], [81]] whereas 4 excluded recent use of only probiotics or antibiotics without specifically excluding the use of other medications [[82], [83], [84], [85]]. The reported *BF* species used were *B. animalis* (*n =* 3), *B. breve* (*n =* 2), *B. longum* (*n =* 2), *B. subtillis* (*n =* 1), and *B. adolescentis* (*n =* 1). Probiotic supplementation duration ranged from 2 to 24 wk, and delivery methods included adding *BF* cultures to a dairy drink or delivering *BF* in a capsule form. All RCTs reported FBG whereas 4 reported HbA1c, so FBG results are summarized here whereas HbA1c results are provided in Appendix B, Supplemental Figures 6, 7.

**TABLE 1:** Summary of the experimental designs and findings of clinical trials

Identifier	Population	ITT sample size (cross-over^1^)	Gender ratio (M/F)	Probiotic species	Duration (wk)	Delivery and dose	Change from baseline	Differences between probiotic and placebo arms
Bernini et al. [81] 2016	Adults with MetS criteria	25 placebo, 26 probiotic	Not reported	*B. animalis* spp. *lactis* nov-HN019	6.4	3.4 × 10e^8^ CFU/mL in 80 mL milk	ND FBG	ND FBG
Culpepper et al. [79] 2019a	Adults with elevated waist circumference	19–20 per group^1^	3/32	*B. subtillis* R0179	6; 4-wk washout	2.5 × 10e^9^ CFU; capsule	—	ND FBG between groups^4^
Culpepper et al. [79] 2019b	Adults with elevated waist circumference	19–20 per group^1^	10/23	*B. animalis* spp. *lactis* B94^2^	6; 4-wk washout	5 × 10e^9^ CFU; capsule	—	ND FBG between groups^5^
Minami et al. [82] 2015	Adults with elevated BMI, most but not all were diabetic	28 placebo, 24 probiotic	11/14 placebo, 6/13 probiotic	*B. breve* B-3^3^	12	5 × 10e^10^ CFU; capsule	ND FBG or glycoalbumin; ↑HbA1c at weeks 4, 8, and 12 compared with baseline in placebo and at weeks 4 and 8 in probiotic	ND FBG or glycoalbumin
Ming et al. [83] 2021	Adults with elevated FBG/OGTT and normal to obese BMI	100 probiotic, 99 placebo	54/45 placebo, 59/41 probiotic	*B. adolescentis*^2^	16	2 × 10e^8^ CFU; capsule	ND FBG, HbA1c, or 2-hr OGTT	ND in FBG, HbA1c, or 2-hr OGTT
Naumova et al. [84] 2020	Adults “undergoing treatment for obesity-related health complications”	8 (single arm)	3/5	*B. longum* spp. *longum* MC-42^2^	2	10e^7^ CFU/mL in 50 mL of dairy drink	↓FBG from baseline to 2 wk	-
Schellekens et al. [47] 2021	Adults with elevated BMI and W:H ratio	74 probiotic,48 placebo	19/29 placebo, 34/40 probiotic	*B. longum* APC1472^2^	12	10e^10^ CFU; capsule	↑FBG in placebo and ↓in probiotic; ↓ HbA1c in both groups	↓FBG in probiotic compared with placebo; ND HbA1c
Stenman et al. [80] 2016	Adults with overweight or obesity and elevated W:H ratio	56 placebo, 48 probiotic	12/44 placebo, 9/39 probiotic	*B. animalis* spp. *lactis* 420^23^	24	10e^10^ CFU; sachet mixed into fruit smoothie	—	ND FBG, HbA1c between placebo or probiotic
Wang et al. [85] 2019	Adults with criteria of MetS	53 (single arm)	26/21	*B. bifidum* TMC3115^2^	3	3 × 10e^10^ CFU/mL; twice daily drink packet	ND FBG	—

Abbreviations: FBG, fasting blood glucose; HbA1c, hemoglobin A1c; ITT, Intention-to-Treat; MetS, metabolic syndrome; ND, no difference; OGTT, oral glucose tolerance test; W:H, waist to hip ratio.

The study identifier, population, sample size (ITT), and ratio of male to female participants are shown along with the probiotic species, duration, delivery method, and dosage. Major findings of glycemic measures are presented in 2 ways; change from baseline in the probiotic arm, and differences between placebo and probiotic arms at the end of the study. Arrows show the direction of significant differences compared with baseline (column second from right) or placebo (furthest right) arms whereas “ND” indicates no significant differences.

1 Denotes a cross-over design; all others were parallel designs. Studies that tested multiple probiotic strains are reported separately with lowercase letters after the study identifier.

2 Indicates that the probiotic was purchased commercially or from a culture collection; no symbol indicates that the probiotic source was unclear.

3 Indicates that the probiotic concentration and viability were verified at least once during the study.

4 No comparison with the placebo group.

5 FBG was higher in *B. animalis* than placebo at baseline; no post-trial comparison to the placebo group.

### Systematic review using clinical trial data

In parallel-design RCTs, significant differences in FBG concentrations between *BF* and placebo arms were reported in only 1 study that recruited 124 adults with obesity, considered as BMI between 28 and 34.9 and waist:hip ratio of ≥0.88 for males and ≥0.83 for females [47]. They found a 4.5% difference in FBG concentrations, from 90.18 ± 9.98 mg/dL in the placebo group compared with 86.04 ± 7.74 mg/dL in the group supplemented with 10e^10^ CFU of *B. longum* APC1472 in a daily capsule. However, this study found no difference in HbA1c after 12 wk of *BF* supplementation. All other studies (*n =* 4) reported no differences in FBG concentrations between *BF* and placebo arms [[80], [81], [82], [83]]. This was accompanied by no differences in 2-h OGTT in 1 study [83].

Two studies reported a reduction in FBG concentrations from baseline values in *BF-*supplemented arms. One study of adults “undergoing treatment for obesity-related health complications” found a significant decrease in FBG concentrations from a median of 97.2–91.8 mg/dL (5.5%) after 2 wk of supplementation with *B. longum*, although 3 were only 8 participants [84]. BMI ranged from 30.0 to 37.0 kg/m^2^ and standard error measurements were not provided which precluded meta-analysis of this study, and the study was also identified to be at a high risk of bias (Supplemental Table 4). A second trial in adults with elevated BMI and waist:hip circumference ratio reported that FBG concentrations significantly increased in the placebo arm from baseline (from 86.58 ± 11.22 to 90.18 ± 9.98 mg/dL, a 4% increase) and decreased in the probiotic arm from baseline (from 89.28 ± 9.29 to 86.04 ± 7.74 mg/dL, a 3.6% decrease) [47]. All other studies (*n =* 4) reported no differences from baseline [[81], [82], [83],^85^].

Three of the RCTs supplemented *B. animalis spp. lactis* with 3 different reported strains for an intervention duration of 6–24 wk, and none observed differences in FBG concentrations between groups [[79], [80], [81]]. Two studies found decreased FBG concentrations from baseline after supplementing different *B. longum* strains for 2 wk [84] or 12 wk [47]. The other 4 studies reported using a strain of *B. subtillis, B. breve*, *B. bifidum*, or *B. adolescentis* (Table 1). Doses ranged from 10^7^ to 10^10^ CFU per dose with some studies delivering 2 doses per day. Probiotic dose seemed less predictive of efficacy; for example, the study with the highest dose of 3 × 10^10^ CFU/mL twice per day found no differences in FBG concentrations after 3 wk of probiotic supplementation [85]. Only 2 of the studies reported verifying that the probiotic remained stable and viable throughout the intervention period [80,82].

### Meta-analysis using clinical trial data

**FIGURE 4:**
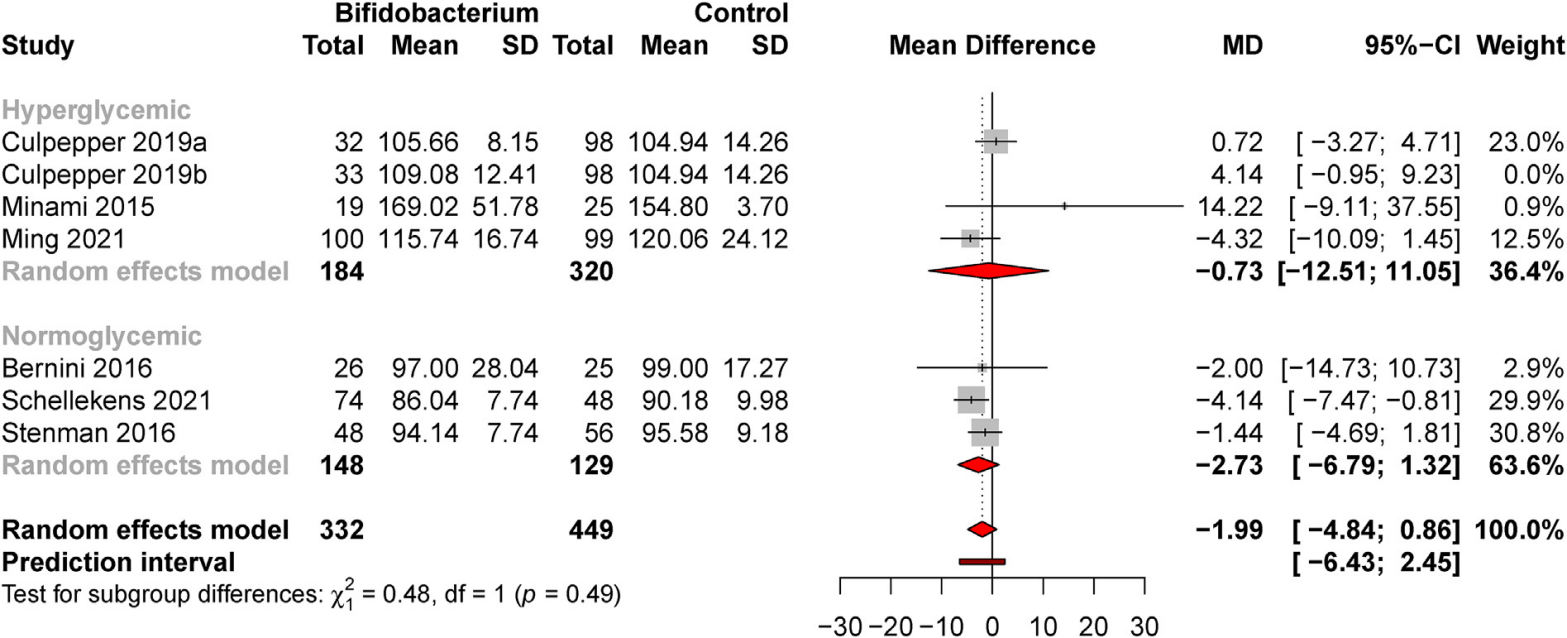
*Bifidobacterium* supplementation does not affect fasting blood glucose in human subjects with hyperglycemia or normoglycemia. A forest plot is shown with subgroup and pooled mean differences of 6 RCTs (with 1 study reporting 2 *Bifidobacterium* strains). One influential study was excluded from the effect size estimates.

Six RCTs comprising 781 subjects were included in the meta-analysis. One cross-over trial tested 2 *BF* species in 2 separate experimental periods that were reported as parallel arms, so it is reported twice and delineated at “Culpepper 2019a” for *B. subtilis* and “Culpepper 2019b” for *B. animalis* (Table 1). All 6 studies reported FBG, whereas 5 reported HbA1c and 6 reported 2-h OGTT; therefore, a meta-analysis is reported for FBG, and a meta-analysis for HbA1c is provided in Appendix B. One influential study was removed, which did not affect the pooled or subgroup effect size estimates. A subgroup analysis compared elevated FBG concentrations (baseline mean FBG >100 mg/dL) to subjects with normoglycemia. There was no effect of *BF* supplementation on FBG in the pooled dataset [MD = −1.99 mg/dL (−4.84, 0.86), *t* = −1.80, *P* = 0.13, and *d* = −0.14 (−0.41, 0.14)] or between subgroups (*χ*^2^ = 0.48, *P* = 0.49) (Figure 4). Subgroup heterogeneity was low (normoglycemic *I*^*2*^ = 0.0% and hyperglycemic *I*^*2*^ = 43.9%), and publication bias was minimal (Supplemental Figure 5).

## Discussion

To determine if *BF* probiotic supplementation modulates blood glucose regulation in hosts with obesity, MetS, or T2D, we conducted a meta-analysis and systematic review of animal studies and RCTs. In animal studies when healthy animals were supplemented with *BF*, glycemic metrics (FBG, HbA1c, or OGTT/IPGTT) were not affected by probiotic supplementation, which was confirmed in the meta-analysis. In studies using models of DIO, *BF* supplementation consistently decreased FBG concentrations in HFD-fed C57Bl/6 mice compared with untreated DIO controls [40,42,[47], [48], [49], [50], [51], [52], [53], [54], [55]] with varied results in other mouse and rat studies [46,49,[58], [59], [60], [61], [62], [63], [64], [65]]. No models of MetS reported decreased FBG concentrations with *BF* supplementation [39,[66], [67], [68], [69]]. In models of T2D, the effect of *BF* supplementation on FBG varied, with some studies reporting decreased FBG concentrations with probiotic supplementation [48,54,70,71,[74], [75], [76]] but others reporting no differences in FBG or varied results between different *BF* strains [53,72,73]. The meta-analysis showed an overall significant reduction in FBG concentrations between *BF-*supplemented models of obesity, MetS, or T2D compared with the untreated obese, MetS, or T2D controls (*n =* 16 studies).

Next, to determine the extent to which *BF* modulates FBG, we compared *BF* supplementation in models of obesity, MetS, or T2D to untreated, healthy controls. In DIO models, *BF* supplementation mostly decreased FBG concentrations to levels similar to standard-fed, untreated controls in most studies [40,42,46,47,50,52,56,[62], [63], [64]]; however, a few studies reported that FBG concentrations remained higher in the DIO group or observed strain-specific variation [48,53,55,65]. One study using a model of MetS reported increased FBG concentrations in *BF* supplementation compared with healthy controls [66] whereas 2 reported no differences [68,69]. All 3 reported no differences in FBG concentrations between *BF-*supplemented and untreated animals, suggesting that the healthy animals had similar FBG concentrations to animals with the pathophysiology relevant to MetS. Studies using models of T2D generally reported that *BF* supplementation did not improve FBG concentrations to levels comparable to healthy controls [70,72,73,76,77], although 2 studies reported similar or lower FBG concentrations in the *BF-*supplemented model of T2D compared with healthy controls [74,78]. The meta-analysis of 12 studies found no differences in FBG between *BF-*supplemented models of DIO, MetS, or T2D compared with healthy controls. All studies but 2 were conducted in male animals.

Overall, these findings suggest that *BF* modulates blood glucose in animal models of obesity and some models of T2D but were generally ineffective in lowering FBG concentrations in MetS models. Some variability in the results could not be readily explained by probiotics species, dose, duration, or animal model. The most reported *BF* species, *B. animalis*, *B. bifidum*, and *B. longum*, seemed to be equally effective at reducing FBG concentrations with no clear patterns regarding supplementation dosage or duration. However, inconsistencies in reporting probiotic doses made it difficult to compare between studies. There were also discrepancies within some studies in glycemic metrics; some *BF* species in the same study affected FBG concentrations but not HbA1c or OGTT, or vice versa, and multiple studies reported strain-specific effects. This may support the hypothesis that probiotics exert short-term effects on FBG concentrations without a corresponding drop in HbA1c, a metric of glycemic control over ∼3 mo [1,12,14,86].

The meta-analysis of 6 RCTs found no differences in FBG concentrations between placebo and *BF-*supplemented arms in both a pooled analysis and subgroup comparison of subjects with elevated FBG concentrations or normoglycemia. HbA1c was similarly unaffected by *BF* supplementation. A systematic review of 8 RCTs showed some discrepancies between the meta-analysis and the results reported in the studies. One study reported significant differences in FBG between parallel placebo and *BF-*supplemented arms [47], and 2 reported significant reductions in FBG concentrations from baseline in *BF-*supplemented arms [47,84]. Upon closer inspection, those decreases represented 3%–5% reductions in FBG concentrations that may not be clinically meaningful. These small effect sizes somewhat conflict with existing meta-analyses of multiple probiotic species that found favorable effects of probiotic supplementation on FBG in adults with elevated FBG concentrations or T2D [12,14,15,86], but 2 reviews found no differences in FBG concentrations in probiotic supplementation of adults with MetS [3] or T2D [1]. In the current review, the RCTs targeted human populations with obesity and/or MetS criteria, but the average baseline FBG of studies included in the meta-analysis was 107 ± 24 mg/dL and 4 of the studies had baseline FBG <100 mg/dL indicating that most of the populations were in the lower range of elevated FBG concentrations or were normoglycemic [4,5]. These off-target study populations may partially explain the null effect of *BF* on glycemic metrics, as multispecies probiotics have previously been shown to have the greatest effects on FBG in T2D [9,13,87] or adjuvant to antidiabetic drugs [54,65,71]. A recent meta-analysis found a greater reduction in HbA1c when prebiotics were used alone or in conjunction with probiotics compared with probiotic-only supplementation [86]. Results from our meta-analysis and systematic review of animal studies agreed with this hypothesis because *BF* had no effect on FBG in healthy animals and exerted varied effects in obese, MetS, and T2D animal models. However, although *B. animalis* was generally effective in animal studies, supplementation with different *B. animalis* strains did not affect FBG in 3 RCTs. Dosage and intervention duration could not readily explain this discrepancy. Taken together, these findings represent translational gaps and may partially explain why *BF* supplementation failed to have a clinically meaningful effect in the RCTs.

The proposed hypotheses by which *BF* improves blood glucose vary and are not fully understood. Some propose that *BF* alters the composition of gut microbiota to remediate gut dysbiosis associated with metabolic disease [80,88]. Others support claims that *BF* competitively excludes pathogenic bacteria at the enterocyte interface to reduce bacterial translocation to the bloodstream [19,20,22]. Anti-inflammatory properties may play a role, as *BF* has been shown to decrease oxidative stress and reduce inflammatory cytokine production from monocytes [13,89]. These putative mechanisms suggest that *BF* may be involved in improving gut permeability and subsequently mitigating inflammation rather than directly interacting with insulin or glucose signaling pathways. Thus, these conflicting findings are not surprising because it is entirely possible that there are interactions between probiotic consumption and host diet, genotype, or environment that create nuances that are difficult to detect in broad meta-analyses. However, it is also possible that multispecies probiotic blends are more effective, given the complex ecologic interactions that govern the gut microbiome and the strength of findings in large meta-analyses of multispecies probiotic blends [9,15,22]. *BF* may also be more effective in lowering blood glucose when delivered in conjunction with prebiotics [86] or adjuvant to antidiabetic drugs [54,65]. Ultimately, more research is needed to fully understand the host-microbe interactions of individual probiotic strains and how they may interact in multistrain formulations or with other pharmaceuticals.

The animal experiments in this review were performed almost exclusively in male animals, but all human RCTs included female and male subjects. The knowledge gained in animal studies of probiotic interactions with host male physiology may not extend to females. The National Institutes of Health published the “Sex as a Biological Variable” policy in 2016 [90], yet in this review, female animals were used in only 2 of the 40 published animal studies. This represents a translational gap and could further explain why *BF* was efficacious in preclinical but not human populations. To better understand female physiology, female animals must be considered in experimental design.

Several factors were noted during the risk of bias assessments and data abstraction that raised concerns of study quality. Several preclinical studies isolated and cultured *BF* strains from stool samples collected in-house rather than buying commercially supplied probiotics or strains from a culture collection (see Supplemental Table 5, Table 1). Other preclinical and clinical studies did not clearly state the source or preparation method of probiotic formulations. This may present a safety concern; though most probiotics are granted Generally Recognized as Safe status by the Federal Food and Drug Administration, they are still considered a food additive and as such, need to be identified correctly and prepared to food-grade standards [23,91,92]. Few studies reported verification of probiotic viability or concentration and even fewer reported using biochemical or sequencing methods to verify the species or strain. No studies reported the results of these tests in the results or supplementary. This raises concerns about the integrity of the test material, especially when the probiotics were isolated in-house or obtained from a culture collection. In RCTs, the study material is often sent home with participants for daily consumption, and only one RCT reported testing the probiotic stability throughout the study to ensure probiotic survival [80]. Furthermore, several preclinical studies reported healthy, standard diet-fed, untreated control animal groups with high-FBG concentrations, even >200 mg/dL. There is no standard consensus for diabetic FBG in mice or rats as there is in humans, and there is conflict in existing protocols; for example, 1 protocol considers 150 mg/dL to be adequate hyperglycemia for T2D whereas another uses 250 mg/dL [27,93]. There may also be methodologic considerations, such as a hyperglycemic response from isoflurane sedation [94]. However, >1 study reported similar FBG in healthy, standard-fed animals and models of DIO or MetS, which brings into question if the generated phenotype appropriately modeled pathophysiology relevant to obesity or MetS. Improving reporting standards, reaching methodologic consensus, and carefully considering the observed, rather than the expected, animal phenotype may improve these conflicts in future research.

Other limitations of this meta-analysis include RCTs with small sample sizes and a high risk of bias. Small clinical trials with large effects are favorably weighted by meta-analysis software, although publication biases were minimal, as observed through funnel plots. Many of the clinical and animal trials were characterized by a high risk of bias and a lack of methodologic reporting. In addition, the sample size of clinical trials did not allow for stratification of *BF* species, treatment duration, or method of probiotic delivery. We have previously shown differences in circulating inflammatory markers in response to varied delivery methods of *B. animalis* spp. *lactis* strain BB-12 in healthy adults [89], thus highlighting the role of the delivery matrix in influencing the immunomodulatory effects of *BF* probiotics. Moreover, species within the *BF* genus have genetic differences that may affect their function and efficacy [16,17]. These nuances should be explored in future clinical trials with improved power.

There is conflicting evidence in recent meta-analyses on the relationship between multispecies probiotics and/or prebiotics and glycemic markers [[1], [2], [3],^9^,^12^,^13^,^15^,^95^]. We hypothesize that this variability might be explained, in part, because of species-specific effects and other confounding interactions, such as improved proliferation of probiotic species in the presence of prebiotics, or using heat-killed instead of live cells in the probiotic formulation. Our targeted meta-analysis and systematic review provide evidence for a favorable effect of *BF* supplementation on FBG in animal models relevant to obesity and T2D, but this finding was not translated to human RCTs. The included RCTs mostly included subjects with normoglycemia or slightly elevated FBG concentrations, which may have contributed to the overall null findings. We identified translational gaps between animal and clinical experiments, such as the paucity of animal research in females, and highlighted concerns regarding studies that had a high risk of bias, isolated and cultured probiotic strains in-house, lacked verification of probiotic concentration, viability, or stability, or reported high-FBG values in healthy animals. These concerns demonstrate an overall lack of reporting appropriate methodologic details that limit the reliability of the study results and represent an overall limitation of this review. Reporting standards must be improved to address these concerns and improve transparency, reproducibility, and rigor in probiotics research. Future research should seek to further control for confounding factors such as intervention duration, probiotic dose, or the use of adjuvant medications in RCTs. *BF* species may have minimal effects on blood glucose in subjects with slightly elevated FBG concentrations, or there may be synergistic interactions with other probiotic species or prebiotic fibers that increase their efficacy. Overall, this systematic review and meta-analysis provides new evidence suggesting a potentially beneficial effect of a single type of probiotic, *BF* species, on markers of glycemic control in animal models and humans with obesity, MetS, or T2D.

### Author contributions

The authors’ responsibilities were as follows – EPVS, EG, CJR: designed the research; EPVS: performed the literature search; EPVS, ZD, JD: conducted literature screening and risk of bias assessments; EPVS: performed the meta-analysis; EPVS, ZD, JD, EG, CJR: wrote the manuscript; and all authors: read and approved the final manuscript.

### Conflict of interest

The authors report no conflicts of interest.

### Funding

This work is supported by the AFRI Predoctoral Fellowship [grant no. 2022-67011-36461] from the USDA National Institute of Food and Agriculture to EPVS. In addition, this work was funded by the National Center for Advancing Translational Sciences of the National Institutes of Health under Award Number TL1TR002016 to ZD. The content is solely the responsibility of the authors and does not necessarily represent the official views of the NIH. The funding agencies had no role in the study conceptualization, design, data collection, analysis, writing, decision to publish, or preparation and submission of the manuscript.

### Data availability

Data described in the manuscript, code book, and analytic code will be made publicly and freely available without restriction at https://github.com/gandalab/bifido-meta-analysis.
