# Supplementary material for: The Effects of Bifidobacterium Probiotic Supplementation on Blood Glucose: A Systematic Review and Meta-Analysis of Animal Models and Clinical Evidence
Source: Adv Nutr. 2023 Nov 2;15(1):100137. doi: 10.1016/j.advnut.2023.10.009 (PMC10831893; doi:10.1016/j.advnut.2023.10.009)
Supplement: Multimedia component 1 [file mmc1.docx]

**The effects of Bifidobacterium probiotic supplementation on blood glucose: a systematic review and meta-analysis of preclinical animal studies and clinical evidence
Van Syoc et al.**

**Online Supplementary Material**

**Supplementary Table 1: Full search terms for each database.** No additional filters or criteria were specified.

| PubMed | ("Bifidobacterium"[Mesh] OR “Bifidobacterium”[TIAB] OR “Bifidobacteria”[TIAB] or “bifido”[TIAB]) AND ("Blood Glucose"[Mesh] OR "Blood Sugar"[TIAB] OR "Blood Sugars"[TIAB] OR "Glycemic Control"[Mesh] OR "Glycemic Control"[TIAB] OR "Glucose Tolerance Test"[Mesh] OR "Glucose Tolerance Test"[TIAB] OR "Glucose Tolerance Tests"[TIAB] OR "Glycated Hemoglobin A"[Mesh] OR "Glycated Hemoglobin A"[TIAB] OR "hba1c"[TIAB] OR "hba"[TIAB] OR "blood glucose"[TIAB] OR "plasma glucose"[TIAB] OR "fasting glucose"[TIAB] OR "fasting plasma glucose"[TIAB] OR "OGTT"[TIAB] OR "oral glucose tolerance"[TIAB] OR “glycated hemoglobin”[TIAB] OR “Glycohemoglobin”[TIAB] OR “Glycohemoglobins”[TIAB] OR “Glycated hemoglobins”[TIAB] OR “Glycosylated Hemoglobin”[TIAB] OR “Glycated Hemoglobin A1c”[TIAB] OR “Glycosylated Hemoglobin A1c”[TIAB] OR “Glycosylated Hemoglobin A1c”[TIAB] OR “Hb A1a-2”[TIAB] OR “Glycated A1a-2 Hemoglobin”[TIAB] OR “Glycated Hemoglobin A”[TIAB] OR “Hb A1a+b”[TIAB] OR “Hb A1c”[TIAB] OR “HbA1”[TIAB] OR “Glycosylated Hemoglobin A”[TIAB] OR “Hb A1”[TIAB] OR “Glycohemoglobin A”[TIAB] OR “Hemoglobin A(1)”[TIAB] OR “Glycosylated A1a-1 Hemoglobin”[TIAB] OR “Hb A1a-1”[TIAB] OR “Glycated A1b Hemoglobin”[TIAB] OR “Hb A1b”[TIAB] OR “Glycosylated A1b Hemoglobin”[TIAB] OR “Fructated Hemoglobins”[TIAB]) |
| --- | --- |
| Web of Science | ("Bifidobacterium" OR “Bifidobacterium” OR “Bifidobacteria” or “bifido”) AND ("Blood Glucose" OR "Blood Sugar" OR "Blood Sugars" OR "Glycemic Control" OR "Glycemic Control" OR "Glucose Tolerance Test" OR "Glucose Tolerance Test" OR "Glucose Tolerance Tests" OR "Glycated Hemoglobin A" OR "Glycated Hemoglobin A" OR "hba1c" OR "hba" OR "blood glucose" OR "plasma glucose" OR "fasting glucose" OR "fasting plasma glucose" OR "OGTT" OR "oral glucose tolerance" OR “glycated hemoglobin” OR “Glycohemoglobin” OR “Glycohemoglobins” OR “Glycated hemoglobins” OR “Glycosylated Hemoglobin” OR “Glycated Hemoglobin A1c” OR “Glycosylated Hemoglobin A1c” OR “Glycosylated Hemoglobin A1c” OR “Hb A1a-2” OR “Glycated A1a-2 Hemoglobin” OR “Glycated Hemoglobin A” OR “Hb A1a+b” OR “Hb A1c” OR “HbA1” OR “Glycosylated Hemoglobin A” OR “Hb A1” OR “Glycohemoglobin A” OR “Hemoglobin A(1)” OR “Glycosylated A1a-1 Hemoglobin” OR “Hb A1a-1” OR “Glycated A1b Hemoglobin” OR “Hb A1b” OR “Glycosylated A1b Hemoglobin” OR “Fructated Hemoglobins”) |
| CAB Direct | ("Bifidobacterium" OR “Bifidobacterium” OR “Bifidobacteria” or “bifido”) AND ("Blood Glucose" OR "Blood Sugar" OR "Blood Sugars" OR "Glycemic Control" OR "Glycemic Control" OR "Glucose Tolerance Test" OR "Glucose Tolerance Test" OR "Glucose Tolerance Tests" OR "Glycated Hemoglobin A" OR "Glycated Hemoglobin A" OR "hba1c" OR "hba" OR "blood glucose" OR "plasma glucose" OR "fasting glucose" OR "fasting plasma glucose" OR "OGTT" OR "oral glucose tolerance" OR “glycated hemoglobin” OR “Glycohemoglobin” OR “Glycohemoglobins” OR “Glycated hemoglobins” OR “Glycosylated Hemoglobin” OR “Glycated Hemoglobin A1c” OR “Glycosylated Hemoglobin A1c” OR “Glycosylated Hemoglobin A1c” OR “Hb A1a-2” OR “Glycated A1a-2 Hemoglobin” OR “Glycated Hemoglobin A” OR “Hb A1a+b” OR “Hb A1c” OR “HbA1” OR “Glycosylated Hemoglobin A” OR “Hb A1” OR “Glycohemoglobin A” OR “Hemoglobin A(1)” OR “Glycosylated A1a-1 Hemoglobin” OR “Hb A1a-1” OR “Glycated A1b Hemoglobin” OR “Hb A1b” OR “Glycosylated A1b Hemoglobin” OR “Fructated Hemoglobins”) |

**Supplementary Table 2: Inclusion and exclusion criteria.**

|  | INCLUSION | EXCLUSION |
| --- | --- | --- |
| Population | - Human subjects or animal trial - Full text in a published journal article in the English language | - In vitro experiments - Preprints, book chapters, thesis reports, or conference abstracts - Reviews and meta-analyses - Journal articles in languages other than English - Study protocols |
| Intervention | - Dietary (or oral gavage) supplementation with *Bifidobacterium* probiotics of any species or strain - Mixture of probiotics that include only *Bifidobacterium* species/strains - RCT design may be parallel or cross-over - Heat-killed, irradiated, or otherwise inactivated probiotics were included | - Probiotic single strain or mixture that includes species/strains other than *Bifidobacterium* - Interventions adjuvant to probiotics other than standard dietary advice - Probiotic species or delivery is not clearly defined |
| Comparison | - In human RCT: placebo arm or baseline comparison - In animal experiment: placebo or positive control (i.e. HFD without probiotics) | - Comparisons that are a different species of probiotic without placebo or baseline comparison |
| Outcome | - Metric of glycemia, including fasting blood glucose, HbA1c, 2-hour OGTT, OGTT AUC - Less frequently described metrics are acceptable, such as fructoseamine | - No metric of glycemia - Glycemia is reported in the Methods but not statistically reported in the Results (i.e., “data not shown”) |

**6Supplementary Figure 1: Modified PRISMA 2020 flowchart**

Records identified from:

Databases (n = 353 PubMed, 392 Web of Science, 405 CAB Direct; 1150 TOTAL)

Records removed *before screening*:

Duplicate records removed (n = 530)

Records removed for other reasons (n = 2) (1 was correction, 1 title was not in English)

Records included in title and abstract screening:

(n = 255)

Reports sought for full-text retrieval:

(n=55)

Reports excluded:

Full text not in English (n=5)

Does not meet eligibility criteria (n=9)

Additional abstracts screened from reference searching of retrieved full texts:

(n=167)

Records excluded:

(n = 285 title was obvious non-fit, 1 conference abstract, 2 study protocols, 75 reviews/book chapters)

Records screened:

(n = 618)

Records removed during abstract screening:

(n = 4 not published in English,

5 were study protocols, 8 were reviews, 238 did not fit inclusion/exclusion criteria)

Reports excluded:

Full text not in English (n=1)

Secondary publication from previously included RCT (n=1)

Does not meet eligibility criteria (n=19)

Total reports sought for full-text retrieval after reference searching:

(n=69)

Studies included in review:

Total (n= 48)

Preclinical (n= 40)

Clinical (n= 8)

**Supplementary Table 3: SYRCLE Risk of Bias assessments for animal experiments.** Y indicates “yes”, N indicates “no”, and U indicates “unclear”. Screening questions: Q1, “Was the allocation sequence adequately generated and applied?”; Q2, “Were the groups similar at baseline or were they adjusted for confounders in the analysis?”; Q3, “Was the allocation to the different groups adequately concealed during?”; Q4, “Were the animals randomly housed during the experiment?”; Q5, “Were the caregivers and/or investigators blinded from knowledge which intervention each animal received during the experiment?”; Q6, “Were animals selected at random for outcome assessment?”; Q7, “Was the outcome assessor blinded?”; Q8, “Were incomplete outcome data adequately addressed?”; Q9, “Are reports of the study free of selective outcome reporting?”; Q10, “Was the study apparently free of other problems that could result in a high risk of bias?”

| IDENTIFIER | Q1 | Q2 | Q3 | Q4 | Q5 | Q6 | Q7 | Q8 | Q9 | Q10 |
| --- | --- | --- | --- | --- | --- | --- | --- | --- | --- | --- |
| ALMADA2021 | Y | U | U | U | U | U | U | Y | Y | Y |
| AN2011 | Y | U | U | U | U | U | U | U | Y | N |
| AOKI2017 | U | N | U | U | U | U | U | U | Y | N |
| BEN2020 | Y | U | U | U | U | U | U | Y | Y | Y |
| BOMHOF2014 | Y | Y | U | U | U | U | U | N | Y | Y |
| CAIMARI2017 | Y | U | U | U | U | U | U | N | Y | Y |
| CANO2013 | Y | U | U | U | U | U | U | Y | N | Y |
| CARRERAS2018 | Y | U | U | U | U | U | U | N | Y | Y |
| CHEN2011 | U | Y | U | U | U | U | U | Y | Y | Y |
| DEABREU2022 | U | N | U | U | U | U | U | Y | Y | N |
| HAO2022 | Y | Y | U | U | U | U | U | Y | Y | Y |
| HORICHU2020 | U | U | U | U | U | U | U | Y | Y | N |
| HSU2021 | Y | Y | U | U | U | U | U | Y | Y | Y |
| HUANG2020 | Y | U | U | U | U | U | U | Y | Y | Y |
| JIANG2022 | U | U | U | U | U | U | U | Y | Y | N |
| KIKUCHI2018 | U | U | U | U | U | U | U | N | Y | N |
| KIM2014 | Y | Y | U | U | U | U | U | Y | Y | Y |
| KONDO2010 | U | U | U | U | U | U | U | U | Y | Y |
| LE2014 | Y | U | U | U | U | U | U | Y | N | N |
| LI2020 | Y | U | U | U | U | U | U | Y | Y | Y |
| LIANG2014 | U | U | U | U | U | U | U | N | Y | N |
| LIM2017 | Y | U | U | U | U | U | U | Y | Y | N |
| MACHADO2021 | U | U | U | U | U | U | U | Y | Y | Y |
| MOUNTS2015 | Y | U | U | U | U | U | U | Y | Y | Y |
| MOYA2016 | Y | U | U | U | U | U | U | Y | Y | N |
| OHNO2004 | U | U | U | U | U | U | U | Y | N | N |
| PLAZA2014 | Y | U | U | U | U | U | U | N | Y | Y |
| QIAN2022 | Y | U | U | U | U | U | U | Y | Y | N |
| RAY2018 | Y | U | U | U | U | U | U | Y | Y | Y |
| REICHOLD2014 | Y | U | U | U | U | U | U | Y | N | Y |
| SALAZAR2014 | U | U | U | U | U | U | U | Y | Y | Y |
| STENMAN2014 | U | N | U | U | U | U | U | N | N | N |
| STENMAN2015 | Y | Y | U | U | U | U | U | Y | Y | Y |
| WANG2015 | Y | U | U | U | U | U | U | Y | Y | Y |
| WANG2020 | U | Y | U | U | U | U | U | Y | Y | Y |
| YDE2021 | U | U | U | U | U | U | U | Y | Y | Y |
| YIN2010 | Y | U | U | U | U | U | U | Y | Y | N |
| ZHANG2020 | Y | Y | U | Y | U | U | U | Y | Y | Y |
| ZHANG2022 | Y | Y | U | U | U | Y | U | Y | N | Y |
| ZHAO2020 | Y | Y | U | U | U | U | U | Y | Y | N |
| ZHAO2022 | Y | U | U | U | U | U | U | Y | Y | Y |
| ZHOU2000 | Y | N | U | U | U | U | U | Y | N | N |
| ZHOU2020a | Y | U | U | U | U | U | U | Y | Y | Y |
| ZHU2018 | Y | U | U | U | U | U | U | N | Y | N |

**Supplementary Table 4: Risk of Bias 2 (RoB 2) bias assessment for human randomized clinical trials.** RoB 2 tool was used for parallel trials or cross-sectional trials (denoted with ^ after study identifier. Two studies had only one intervention arm (denoted with * after study identifier). Each domain is ranked as low risk of bias, some concerns, or high risk of bias.

Risk of bias (ROB) was assessed in the following domains: (1) ROB arising from randomization process; (S – only in crossover trials) ROB arising from period and carryover effects; (2) ROB due to deviations from the intended interventions; (3) ROB due to missing outcome data; (4) ROB in measurement of the outcome; (5) ROB in selection of the reported result; (6) Overall risk of bias.

| Identifier | 1 | S | 2 | 3 | 4 | 5 | 6 |
| --- | --- | --- | --- | --- | --- | --- | --- |
| Bernini 2016 | HIGH | NA | SOME | LOW | LOW | HIGH | **HIGH** |
| Culpepper 2019^ | LOW | LOW | LOW | LOW | HIGH | SOME | **HIGH** |
| Minami 2015 | SOME | NA | LOW | LOW | LOW | SOME | **SOME** |
| Ming 2021 | LOW | NA | LOW | LOW | LOW | LOW | **LOW** |
| Naumova 2015* | NA | NA | HIGH | HIGH | HIGH | HIGH | **HIGH** |
| Schellekens 2021 | LOW | NA | LOW | LOW | LOW | LOW | **LOW** |
| Stenman 2016 | LOW | NA | SOME | LOW | LOW | LOW | **LOW** |
| Wang 2019* | NA | NA | HIGH | SOME | HIGH | LOW | **HIGH** |

**Supplementary Table 5: The experimental designs and results of animal studies included in the meta-analysis and/or systematic review.** Each study is shown with an identifier; the experimental phenotype, animal species, and sample size; the species and dose of Bifidobacterium probiotics; the duration and delivery method; and glycemic results. Studies that tested multiple animal models and/or multiple probiotic strains are shown in multiple rows with one animal model and strain per row, differentiated with superscript letters after the study identifier. Separate studies by the same first author in the same year are differentiated with (a). Asterisks denote studies with female animals, all other studies used male animals or did not specify and are assumed to be male. In the two columns showing results, arrows indicate the direction of statistically significant changes compared to the control group; “ND” indicates no significant differences. The symbol ‘⫮’ after probiotic species indicates that the probiotic was isolated and cultured in-house, while the ‘⫲’ symbol indicates that the probiotic was purchased commercially or from a culture collection; no symbol indicates that the probiotic source was unclear. The symbol ‘Ψ’ indicates that probiotic concentration and viability were verified with plate counting or another appropriate method at least once during the study. Further pertinent details about the experimental design are included in footnotes where necessary.

The experimental models are categorized as: Healthy, DIO (diet-induced obesity), TrG (transgenic species), or STZ-D (streptozotocin-induced model of diabetes mellitus). Similarly, transgenic (TrG) models are denoted as inducing the pathophysiology of MetS (TrG-MetS) or T2D (TrG-T2D).

**Abbreviations:**

DIO, diet-induced obesity; MetS, metabolic syndrome; T2D, type 2 diabetes; TrG, transgenic species; STZ-D, streptozotocin injection to induce diabetes; OG, oral gavage; BG, blood glucose; OGTT, oral glucose tolerance test (presented as area under the curve, OGTT-AUC; or concentration at 2 hours, 2-hr OGTT); IPGTT, intraperitoneal glucose tolerance test (presented as area under the curve, IPGTT-AUC, or concentration at 2 hours, 2-hr IPGTT); CFU, colony-forming unit; ND, no difference

| 1. *Bifidobacterium* supplementation in healthy animals | | | | | |
| --- | --- | --- | --- | --- | --- |
| Identifier | **Model**  **(species, per-group n)** | ***Bifidobacterium* species, dose** | **Duration & delivery** | **Probiotic vs healthy control** |  |
| Caimari 2017 | Healthy  (Wistar, 10) | *B. animalis* spp *lactis* CECT8145 (heat-killed),  10e^10^ CFU⫲Ψ | 12 weeks in chow | ↑FBG |  |
| Cano 2013 | Healthy  (C57Bl/6, 6) | *B. pseudocatenulatum* CECT7765,  5x10e^8^ CFU⫮Ψ | 7 weeks OG | ND FBG or OGTT-AUC |  |
| Huang 2020 | Healthy  (Institute of Cancer Research mice, 8) | *B. longum* spp *longum* OLP-01,  1.03x10e^10^ CFU/kg⫮ | 6 weeks OG | ND FBG |  |
| Machado 2021 | Healthy  (Swiss, 8) | *B. longum,*  50 billion bacteria/kg | 4 weeks OG | ND IPGTT-AUC |  |
| Moya-Pérez 2015 | Healthy  (C57Bl/6, 10) | *B. psuedocatenulatum* CECT7765,  10e^9^ CFU⫮Ψ | 14 weeks OG | ND FBG |  |
| Reichold 2014 | Healthy  (C57Bl/6, 4-6) | *B. adolescentis* ATCC 15705,  5x10e^7^ CFU⫮ | 12 weeks in drinking water | ND FBG |  |
| Salazar 2014^a^ | Healthy  (Wistar, 8) | *B. longum* IPLA-E44,  10e^9^ CFU⫲ | 3.4 weeks OG | ND FBG |  |
| Salazar 2014^b^ | Healthy  (Wistar, 8) | *B. animalis* spp *lactis* IPLA-R1,  10e^9^ CFU⫲ | 3.4 weeks OG | ND FBG |  |
| Schellekens 2021 | Healthy  (C57Bl/6, 8-10) | *B. longum* APC1472,  2x10e^8^ CFU/mL⫮Ψ | 16 weeks in drinking water | ND OGTT-AUC |  |
| Wang 2015 | Healthy  (BALB/c, 10)* | *B. bifidum* WBlNO1,  5x10e^10^ CFU⫮ | 4 weeks, delivery unclear | ND FBG |  |
| 1. *Bifidobacterium* supplementation in obese, MetS, T2D animals | | | | | |
| Identifier | **Model**  **(species, per-group n)** | ***Bifidobacterium* species, dose** | **Duration; delivery** | **Probiotic vs healthy control** | **Probiotic vs untreated metabolic disease** |
| Almada 2015 | DIO  (Wistar, 7) | *B. animalis* spp *lactis* BB-12 (irradiated),  10e^8^ CFU/day⫲ | 2.1 weeks added to pasta | ↓FBG | ND FBG |
| An 2011 | DIO  (Sprague-Dawley, 12) | Mixture: *B. pseudocatenulatum* SPM 1204, *B. longum* SPM 1205, *B. longum* SPM 1207, 10e^8^-10e^9^ CFU | 5 weeks OG | ND FBG | ↓FBG |
| Aoki 2017^a^ | DIO  (C57Bl/6J, 11-14) | *B. animalis* spp *lactis* GCL2505,  10e^9^ CFU | 7 weeks OG | ↑OGTT-AUC | ↓OGTT-AUC |
| Aoki 2017^b^ | DIO  (C57Bl/6J, 11-14) | *B. animalis* spp *lactis* GCL2505,  10e^9^ CFU | 7 weeks OG | - | ↓OGTT-AUC |
| Aoki 2017^c^ | DIO  (C57Bl/6J, 11-14) | *B. longum* spp *longum* JCM1217,  10e^9^ CFU | 7 weeks OG | - | ND OGTT-AUC |
| Aoki 2017^d^ | TrG-T2D  (*ob/ob*, 10) | *B. animalis* spp *lactis* GCL2505,  10e^9^ CFU | 7 weeks OG | - | ↓OGTT-AUC |
| Ben 2020 | TrG-T2D  (Tsumura Suzuki obese diabetes mice^[[1]](#footnote-1)^, 5) | *B. longum* BR-108 (autoclaved),  3.4x10e^12^ cell/g administered at 50, 100, or 150 mg/kg⫲ | 4.3 weeks OG | ND FBG & 2-hr OGTT in 50, 100, 150 mg/kg | ↓FBG & 2-hr OGTT in 100 and 150 mg/kg (ND 50 mg/kg) |
| Bomhof 2014 | DIO  (Sprague-Dawley, 10) | *B. animalis* spp *lactis* BB-12,  10e^10^ CFU⫲Ψ | 8 weeks in chow | - | ↓2-hr OGTT |
| Caimari 2017 | DIO-MetS^[[2]](#footnote-2)^  (Wistar, 10) | *B. animalis* spp *lactis* CECT8145,  10e^10^ CFU⫲Ψ | 12 weeks in chow | - | ND FBG |
| Cano 2013 | DIO  (C57Bl/6, 6) | *B. pseudocatenulatum* CECT7765,  5x10e^8^ CFU⫮Ψ | 7 weeks OG | ND FBG & OGTT-AUC | ↓FBG & OGTT-AUC |
| Carreras 2018 | TrG-MetS  (obese Zucker, 10) | *B. animalis* spp *lactis* CECT8145,  10e^10^ CFU⫲ | 12 weeks in drinking water | ↑FBG | ND FBG |
| Chen 2011 | DIO-MetS.  (Wistar, 10) | *B. longum* CGMCC No. 2017,  2x10e^9^ CFU⫲ | 12 weeks OG | ND FBG | ND FBG |
| De Abreu Ribeiro Pereira 2022 | DIO  (Swiss, 6) | *B. animalis* spp *lactis*,  10e^9^ CFU/day⫲Ψ | 4.3 weeks OG | ND FBG | ND FBG |
| Hao 2022 | DIO-STZ-D  (Kunming mice, 8) | *B. longum* spp *longum* BL21,  0.2 mL of 5x10e^9^ CFU/mL⫲ | 6 weeks OG | ↑FBG & OGTT-AUC, ND 2hr-OGTT | ↓FBG, 2hr-OGTT, & OGTT-AUC |
| Horiuchu 2022^a^ | DIO  (C57Bl/6J, 10) | *B. animalis* spp *lactis* GCL2505,  10e^9^ CFU⫲ | “6-7 weeks” OG | - | ↓OGTT-AUC |
| Horiuchu 2022^b^ | DIO  (HFD-fed *Gpr43^-/-^*, 7) *^[[3]](#footnote-3)^* | *B. animalis* spp *lactis* GCL2505,  10e^9^ CFU⫲ | “6-7 weeks” OG | - | ND OGTT-AUC |
| Hsu 2021 | TrG-T2D  (*db/db*, 6) | *B. longum* OLP-01,  1.03x10e^10^ CFU/kg/day⫮ | 8 weeks OG | ↓OGTT-AUC & FBG | - |
| Jiang 2022 | DIO  (C57Bl/6J, 10) | *B. longum* 070103,  10 mL/kg (CFU not described)⫮ | 13 weeks in chow | ND FBG & OGTT-AUC | ↓FBG & OGTT-AUC |
| Kim 2014 | TrG-T2D  (KK.Cg-A^y/+^, 7)^[[4]](#footnote-4)^ | *B. lactis* HY8101,  1.67x10e^9^ CFU/g feed⫮ | 6 weeks in chow | - | ↓FBG & 2-hr OGTT |
| Kondo 2010 | DIO  (C57Bl/6J, 6) | *B. breve* B-3,  10e^8^ or 10e^9^ CFU | 8 weeks in chow | - | ↓FBG in both doses |
| Le 2014 | DIO  (Swiss-Webster, 6) | Mixture: *B. bifidum* ATCC 15700, *B. longum*, *B. infantis,* *B. animalis*,  10e^9^ of each | 5 weeks OG | - | ↓2hr-OGTT & OGTT-AUC |
| Li 2020^a^ | DIO (C57Bl/6J, 15) | *B. animalis* spp *lactis* A12,  10e^9^ CFU⫮ | 10 weeks in chow | ↑OGTT-AUC | ↓OGTT-AUC |
| Li 2020^b^ | DIO  (C57Bl/6J, 15) | *B. animalis* spp *lactis* BB12,  10e^9^ CFU⫮ | 10 weeks in chow | ND OGTT-AUC | ↓OGTT-AUC |
| Lim 2017 | DIO  (C57Bl/6, 8) | *B. adolescentis* IM 38 (KCCM 11807),  10e^9^ CFU⫲ | 6 weeks OG | ND FBG | ND FBG |
| Machado 2021 | DIO  (Swiss, 8) | *B. longum*,  50 billion bacteria/kg | 4 weeks OG | ND IPGTT-AUC | ↓FBG & IPGTT-AUC |
| Mounts 2015 | TrG-MetS  (obese Zucker, 3)* | *B. longum* BB36,  0.1% (CFU not provided) | 14.3 weeks in chow | - | ND FBG |
| Moya-Pérez 2015 | DIO  (C57Bl/6, 10) | *B. psuedocatenulatum* CECT7765,  10e^9^ CFU⫮Ψ | 14 weeks OG | ND FBG | ↓FBG |
| Ohno 2004^a^ | TrG-T2D  (obese Wistar, 6) | *B. bifidum* G9-1,  2x10e^11^ CFU/g⫲ | 3 weeks in chow | ↑FBG | ND FBG |
| Ohno 2004^b^ | TrG-T2D  (KK-A^y^ mice, 6) | *B. bifidum* G9-1,  2x10e^11^ CFU/g⫲ | 2 weeks in chow | - | ↓FBG |
| Plaza-Diaz 2014 | TrG-MetS  (obese Zucker, 10) | *B. breve* CNCM I-4035,  10e^10^ CFU | 4.3 weeks OG | ND FBG | ND FBG |
| Qian 2022^a^ | DIO-STZ-D^[[5]](#footnote-5)^  (C57Bl/6, 6) | *B. adolescentis* FJSSZ3M10,  2x10e^8^ CFU/ml⫲ | 12 weeks OG | ↑OGTT-AUC | ND OGTT-AUC |
| Qian 2022^b^ | DIO-STZ-D  (C57Bl/6, 6) | *B. adolescentis* FGSZY8M4 (CCFM1108),  2x10e^8^ CFU/ml⫲ | 12 weeks OG | ↑OGTT-AUC | ↓OGTT-AUC |
| Qian 2022^c^ | DIO-STZ-D  (C57Bl/6, 6) | *B. adolescentis* FNNFQ26M1,  2x10e^8^ CFU/ml⫲ | 12 weeks OG | ↑OGTT-AUC | ND OGTT-AUC |
| Qian 2022^d^ | DIO-STZ-D  (C57Bl/6, 6) | *B. adolescentis* FGSYC30M5,  2x10e^8^ CFU/ml⫲ | 12 weeks OG | ↑OGTT-AUC | ND OGTT-AUC |
| Qian 2022^e^ | DIO-STZ-D  (C57Bl/6, 6) | *B. adolescentis* FXJKS34M4,  2x10e^8^ CFU/ml⫲ | 12 weeks OG | ↑OGTT-AUC | ND OGTT-AUC |
| Qian 2022^f^ | DIO-STZ-D  (C57Bl/6, 6) | *B. adolescentis* FHNFQ41M3,  2x10e^8^ CFU/ml⫲ | 12 weeks OG | ↑OGTT-AUC | ↓OGTT-AUC |
| Qian 2022^g^ | DIO-STZ-D  (C57Bl/6, 6) | *B. adolescentis* FXJCJ50M3,  2x10e^8^ CFU/ml⫲ | 12 weeks OG | ↑OGTT-AUC | ND OGTT-AUC |
| Qian 2022^h^ | DIO-STZ-D  (C57Bl/6, 6) | *B. adolescentis* HuNan112 (CCFM1261) , 2x10e^8^ CFU/ml⫲ | 12 weeks OG | ↑OGTT-AUC | ↓OGTT-AUC |
| Qian 2022^i^ | DIO-STZ-D  (C57Bl/6, 6) | *B. bifidum* FJSSZ5M8,  2x10e^8^ CFU/ml⫲ | 12 weeks OG | ↑OGTT-AUC | ND OGTT-AUC |
| Qian 2022^j^ | DIO-STZ-D  (C57Bl/6, 6) | *B. bifidum* FSDJN705,  2x10e^8^ CFU/ml⫲ | 12 weeks OG | ↑OGTT-AUC | ND OGTT-AUC |
| Qian 2022^k^ | DIO-STZ-D  (C57Bl/6, 6) | *B. bifidum* FXJCJ9M10,  2x10e^8^ CFU/ml⫲ | 12 weeks OG | ↑OGTT-AUC | ND OGTT-AUC |
| Qian 2022^l^ | DIO-STZ-D  (C57Bl/6, 6) | *B. bifidum* JSWX19M5,  2x10e^8^ CFU/ml⫲ | 12 weeks OG | ↑OGTT-AUC | ND OGTT-AUC |
| Qian 2022^m^ | DIO-STZ-D  (C57Bl/6, 6) | *B. bifidum* AHWH21M3,  2x10e^8^ CFU/ml⫲ | 12 weeks OG | ↑OGTT-AUC | ND OGTT-AUC |
| Qian 2022^n^ | DIO-STZ-D  (C57Bl/6, 6) | *B. bifidum* FHNFQ23M2,  2x10e^8^ CFU/ml⫲ | 12 weeks OG | ↑OGTT-AUC | ND OGTT-AUC |
| Qian 2022^o^ | DIO-STZ-D  (C57Bl/6, 6) | *B. bifidum* FHNFQ26M7 (CCFM1165),  2x10e^8^ CFU/ml⫲ | 12 weeks OG | ↑OGTT-AUC | ND OGTT-AUC |
| Qian 2022^p^ | DIO-STZ-D  (C57Bl/6, 6) | *B. bifidum* JSWX267,  2x10e^8^ CFU/ml⫲ | 12 weeks OG | ↑OGTT-AUC | ND OGTT-AUC |
| Ray 2018 | DIO  (Albino mice, 10) | B. sp MKK4,  10e^9^ CFU⫮ | 8 weeks, delivery mode unknown | ND FBG | ↓FBG |
| Reichold 2014 | DIO-MetS  (C57Bl/6, 4-6) | *B. adolescentis* ATCC 15705,  5x10e^7^ CFU⫮ | 12 weeks in drinking water | ND FBG | - |
| Schellekens 2021 | DIO  (C57Bl/6, 8-10) | *B. longum* APC1472,  2x10e^8^ CFU/mL⫮Ψ | 16 weeks in drinking water | ND OGTT-AUC | ↓OGTT-AUC |
| Stenman 2014^a^ | DIO  (C57Bl/6J, 10) | *B. lactis* 420 ATCC:SD6685,  10e^9^ CFU | 6 weeks OG | ↑90min-IPGTT | ↓90min-IPGTT |
| Stenman 2014^b^ | DIO-T2D^[[6]](#footnote-6)^  (C57Bl/6J, 5-10) | *B. lactis* 420 ATCC:SD6685,  10e^9^ CFU | 6 weeks OG | ND 90-min IPGTT | ND 90-min IPGTT |
| Stenman 2015 | DIO-T2D^[[7]](#footnote-7)^  (C57Bl/6J, 10) | *B. animalis* spp *lactis* 420,  10e^9^ CFU⫲ | 4 weeks OG | - | ↓IPGTT-AUC & FBG |
| Wang 2020^a^ | DIO-STZ-D  (C57Bl/6J, 8) | *B. adolescentis* CCFM 1061 (“N3”),  0.2 mL of 5x10e^9^ CFU/mL⫲ | 5 weeks, OG 6 days/week | ↑FBG, OGTT-AUC, & HbA1c | ↓FBG, OGTT-AUC, & HbA1c |
| Wang 2020^b^ | DIO-STZ-D  (C57Bl/6J, 8) | *B. adolescentis* CCFM 1062 (“7-2”),  0.2 mL of 5x10e^9^ CFU/mL⫲ | 5 weeks, OG 6 days/week | ↑FBG, OGTT-AUC, & HbA1c | ↓FBG, OGTT-AUC, & HbA1c |
| Wang 2020^c^ | DIO-STZ-D  (C57Bl/6J, 8) | *B. adolescentis* ZJ25,  0.2 mL of 5x10e^9^ CFU/mL⫲ | 5 weeks, OG 6 days/week | ↑FBG, OGTT-AUC, & HbA1c | ND FBG or HbA1c, ↓OGTT-AUC |
| Wang 2020^d^ | DIO-STZ-D  (C57Bl/6J, 8) | *B. bifidum* CCFM 1063 (“M2”),  0.2 mL of 5x10e^9^ CFU/mL⫲ | 5 weeks, OG 6 days/week | ↑FBG & OGTT-AUC, ND HbA1c | ↓FBG, OGTT-AUC, & HbA1c |
| Wang 2020^e^ | DIO-STZ-D  (C57Bl/6J, 8) | *B. bifidum* 7-05,  0.2 mL of 5x10e^9^ CFU/mL ⫲ | 5 weeks, OG 6 days/week | ↑FBG, OGTT-AUC, & HbA1c | ND FBG, OGTT-AUC, or HbA1c |
| Wang 2020^f^ | DIO-STZ-D  (C57Bl/6J, 8) | *B. bifidum* 35,  0.2 mL of 5x10e^9^ CFU/mL⫲ | 5 weeks, OG 6 days/week | ↑FBG, OGTT-AUC, & HbA1c | ↓FBG, ND OGTT-AUC or HbA1c |
| Yde 2021 | DIO  (C57Bl/6J, 40) | *B. animalis* spp *lactis* 420,  10e^9^ CFU | 9 weeks OG | - | ND IPGTT-AUC |
| Yin 2010^a^ | DIO  (Sprague-Dawley, 8) | *B.* L66-5 (no species given),  0.4 mL of 10e^8^ CFU/mL⫮Ψ | 6 weeks OG | ND FBG | ND FBG |
| Yin 2010^b^ | DIO  (Sprague-Dawley, 8) | *B.* L75-4 (no species given),  0.4 mL of 10e^8^ CFU/mL⫮Ψ | 6 weeks OG | ND FBG | ND FBG |
| Yin 2010^c^ | DIO  (Sprague-Dawley, 8) | *B.* M13-4 (no species given),  0.4 mL of 10e^8^ CFU/mL⫮Ψ | 6 weeks OG | ND FBG | ND FBG |
| Yin 2010^d^ | DIO  (Sprague-Dawley, 8) | *B.* FS31-12 (no species given),  0.4 mL of 10e^8^ CFU/mL⫮Ψ | 6 weeks OG | ND FBG | ND FBG |
| Zhang 2020^a^ | DIO-STZ-D^[[8]](#footnote-8)^  (Sprague-Dawley, 6) | *B. animalis* 01,  10e^9^ CFU | 16 weeks OG | - | ↓HbA1c & OGTT-AUC |
| Zhang 2020^b^ | DIO-STZ-D^[[9]](#footnote-9)^  (Sprague-Dawley, 6) | *B. animalis* 01,  10e9 CFU | 10 weeks OG | - | ↓HbA1c & OGTT-AUC |
| Zhao 2020 | DIO-STZ-D  (C57Bl/6J, 8) | *B. longum* DD98,  5x10e^9^ CFU/mLΨ | 2 weeks OG | ↑OGTT-AUC, FBG, & HbA1c | ↓OGTT-AUC, FBG, & HbA1c |
| Zhao 2020(a) | DIO  (C57Bl/6J. 8) | *B. longum* DD98,  5x10e^9^ CFU/mLΨ | 6 weeks OG | ↑FBG & OGTT-AUC | ↓FBG &OGTT-AUC |
| Zhu 2018^a^ | DIO  (Sprague-Dawley, 6) | *B. longum* C-1 A4,  2mL of 3x10e^9^ CFU/mL⫮ | 12 weeks OG | ND FBG, ↑OGTT-AUC | ↓FBG, ND OGTT-AUC |
| Zhu 2018^b^ | DIO  (Sprague-Dawley, 6) | *B. longum* K2,  2mL of 3x10e^9^ CFU/mL⫮ | 12 weeks OG | ND FBG, ↑OGTT-AUC | ↓FBG & OGTT-AUC |
| Zhu 2018^c^ | DIO  (Sprague-Dawley, 6) | *B. breve* R2,  2mL of 3x10e^9^ CFU/mL⫮ | 12 weeks OG | ↑FBG, ND OGTT-AUC | ND FBG & OGTT-AUC |
| Zhu 2018^d^ | DIO  (Sprague-Dawley, 6) | *B. breve* S13,  2mL of 3x10e^9^ CFU/mL⫮ | 12 weeks OG | ↑FBG, OGTT-AUC | ND FBG, ↑OGTT-AUC |
| Zhu 2018^e^ | DIO  (Sprague-Dawley, 6) | *B. adolescentis* Z25,  2mL of 3x10e^9^ CFU/mL⫮ | 12 weeks OG | ND FBG, OGTT-AUC | ↓FBG & OGTT-AUC |
| Zhu 2018^f^ | DIO  (Sprague-Dawley, 6) | *B. bifidum* W25,  2mL of 3x10e^9^ CFU/mL⫮ | 12 weeks OG | ND FBG, ↑OGTT-AUC | ↓FBG & OGTT-AUC |
| Zhu 2018^g^ | DIO  (Sprague-Dawley, 6) | *B. bifidum* F35,  2mL of 3x10e^9^ CFU/mL⫮ | 12 weeks OG | ↑FBG, OGTT-AUC | ND FBG & OGTT-AUC |

**Supplementary Figure 2: Publication bias in *Bif* supplementation in healthy animals.**

**
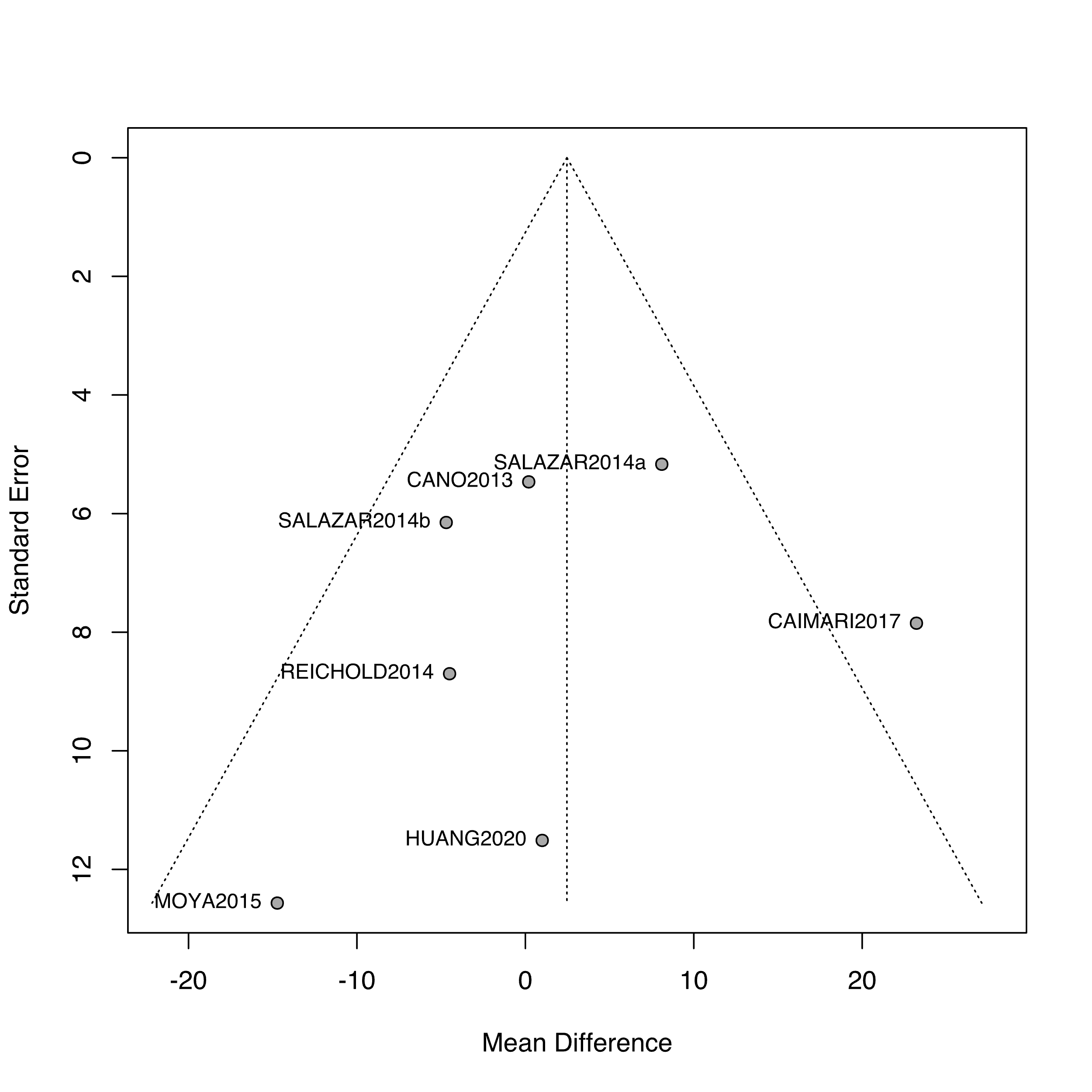
**

**Supplementary Figure 3: Publication bias in *Bif* supplementation in animals with metabolic disease.**

**
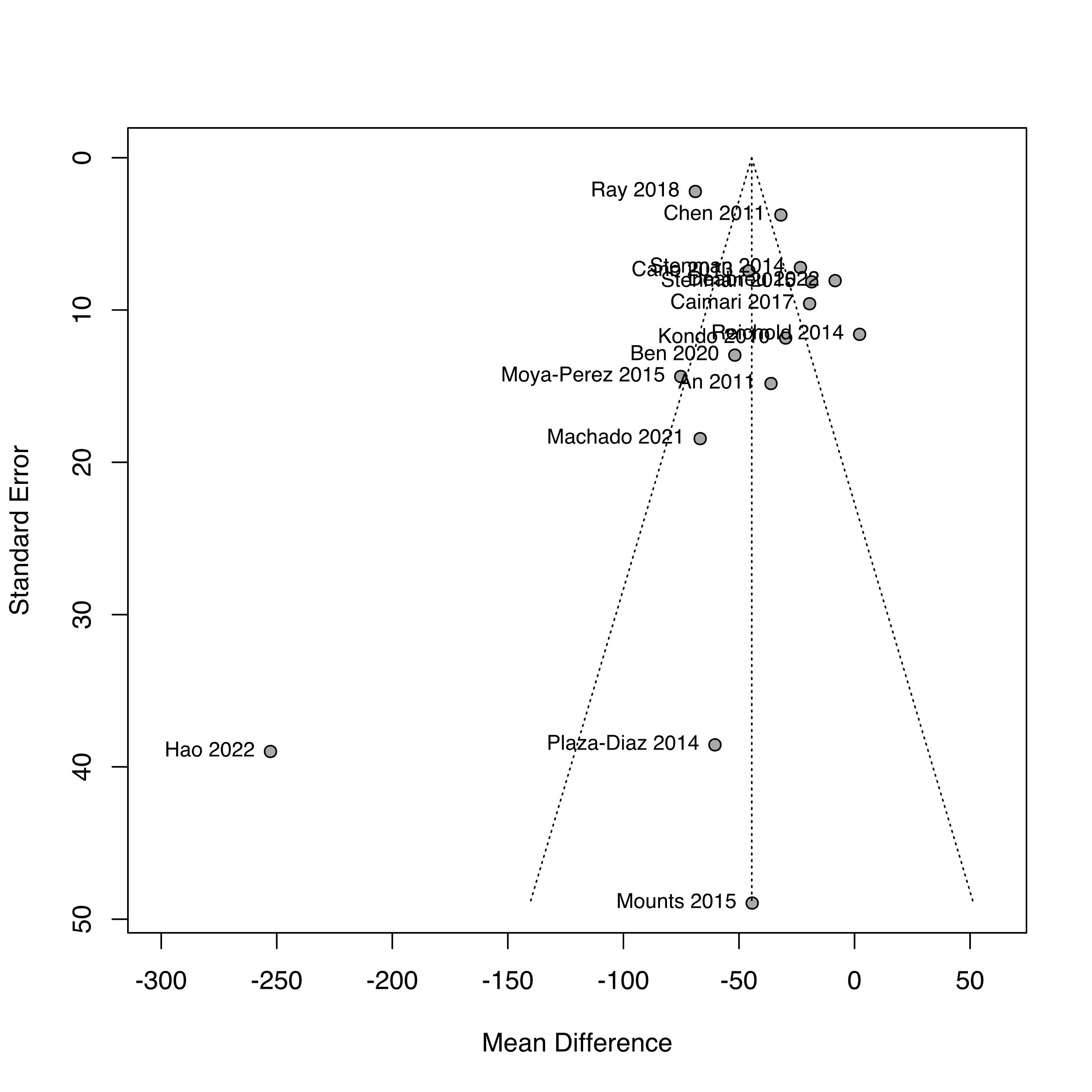
**

**
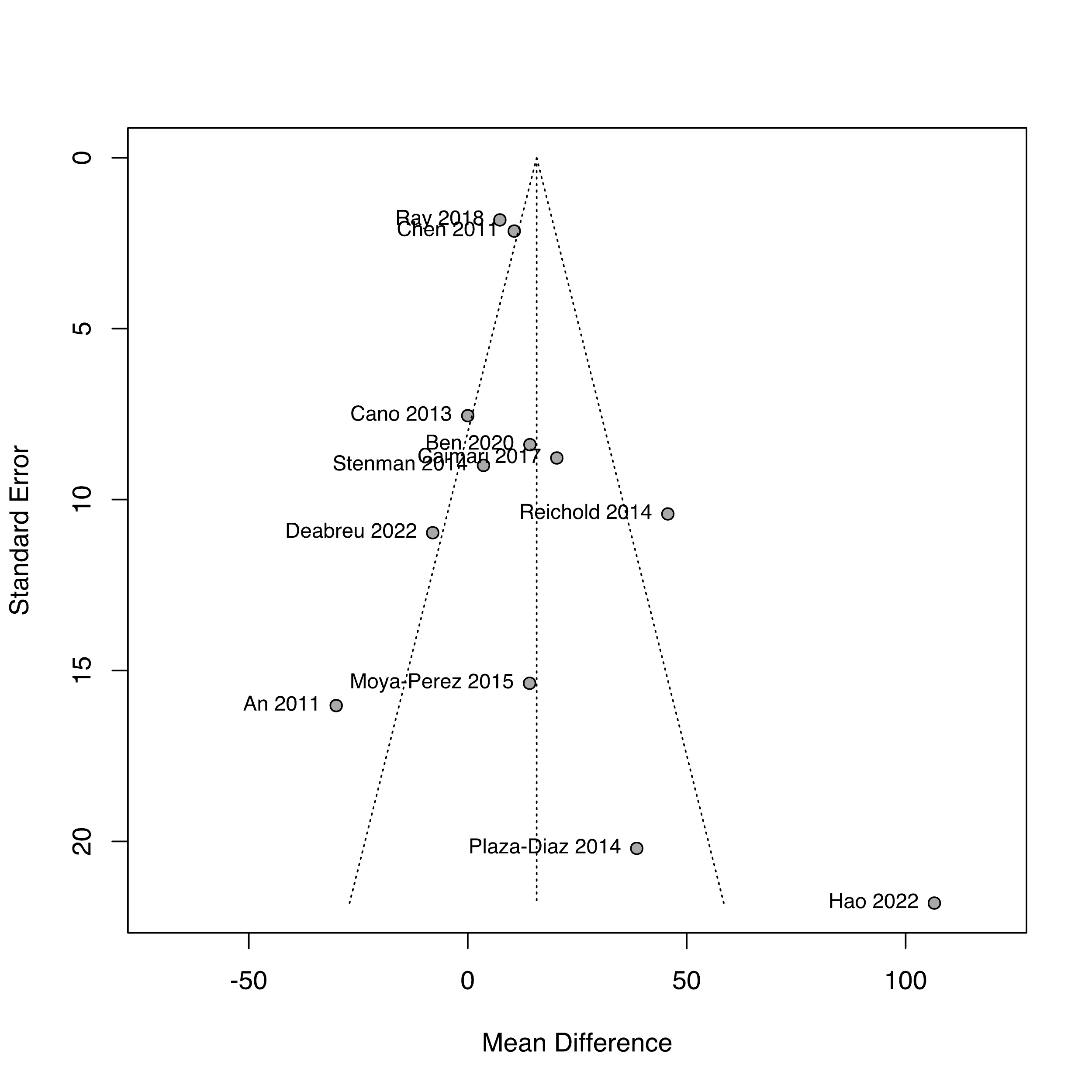
Supplementary Figure 4: Publication bias in *Bif* supplementation in metabolic disease compared to healthy animals.**

**Supplementary Figure 5: Publication bias in the clinical meta-analysis of fasting blood glucose.**

**
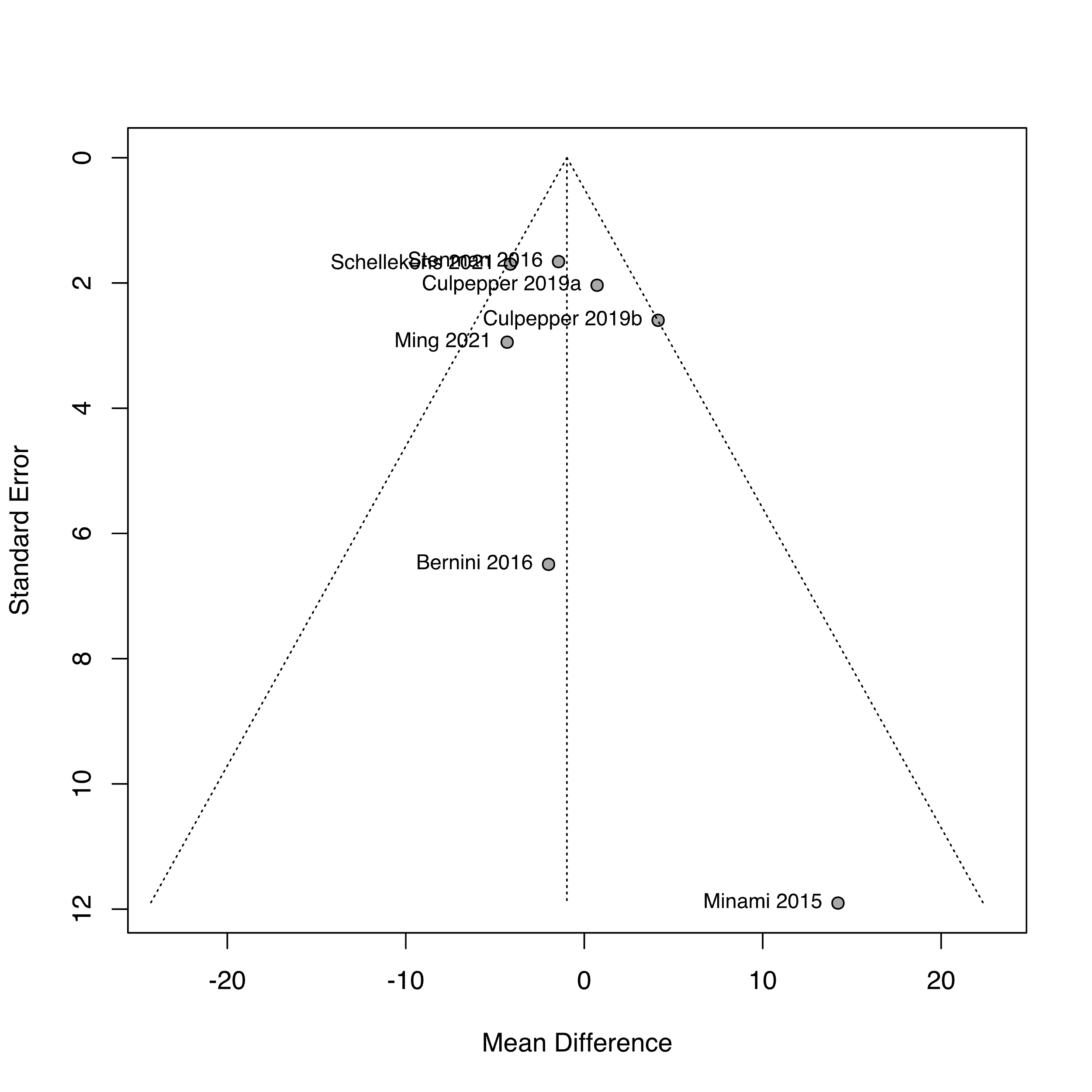
**

**Supplementary Figure 6: *Bifidobacterium* supplementation does not affect hemoglobin A1c in obese subjects.** A forest plot shows the mean difference estimates in 4 studies. One influential study was removed from the effect estimates.

**
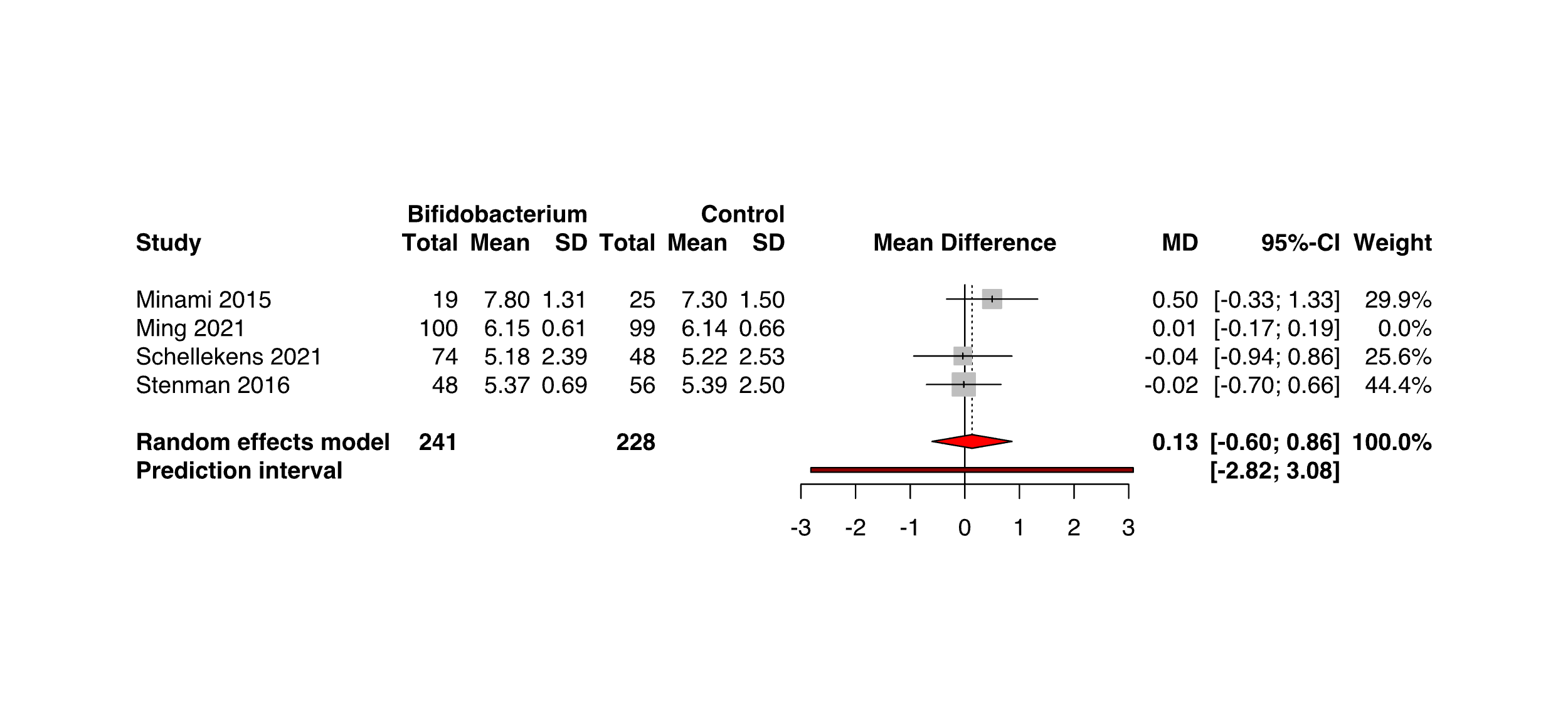
**

**Supplementary Figure 7: Publication bias in the clinical meta-analysis of HbA1c
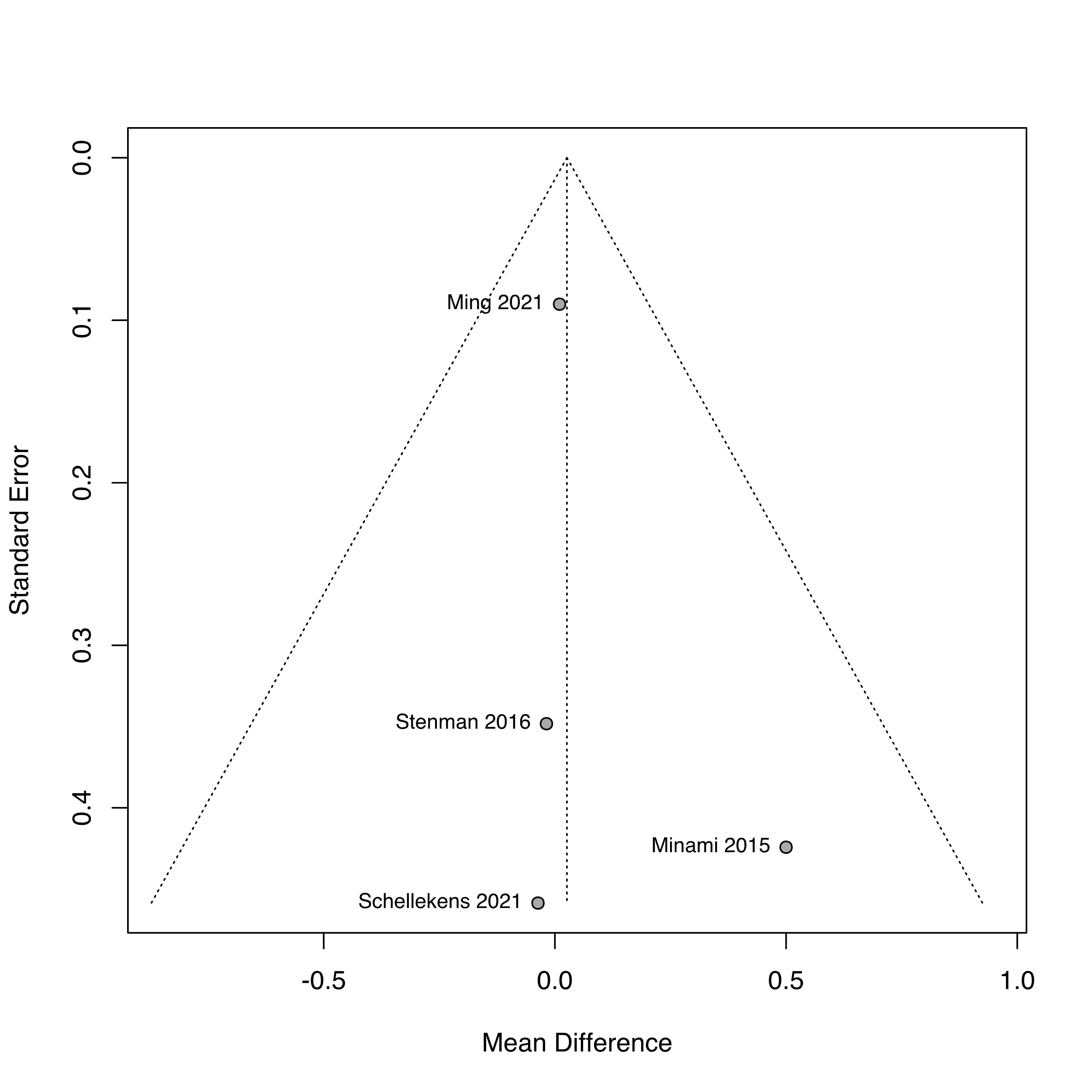
**

1. Healthy controls were Tsumura Suzuki non-obese mice [↑](#footnote-ref-1)
2. “Cafeteria diet” comprised of bacon, biscuit with pate, biscuit with cheese, muffins, carrots, milk with sugar added to standard chow [↑](#footnote-ref-2)
3. G protein-coupled receptor that is activated by short chain fatty acids [↑](#footnote-ref-3)
4. Transgenic non-insulin-dependent diabetes model; C57Bl/6 mice were healthy controls [↑](#footnote-ref-4)
5. Probiotic supplementation and high fat diet-fed for 7 weeks, STZ injection, then additional 5 weeks of probiotic supplementation (for all Qian 2022) [↑](#footnote-ref-5)
6. T2D induced with 72% fat ketogenic diet [↑](#footnote-ref-6)
7. T2D induced with 72% fat ketogenic diet [↑](#footnote-ref-7)
8. “Prevention” group, probiotics given 6 weeks before STZ injections and continuing for 10 weeks after [↑](#footnote-ref-8)
9. “Therapeutic” group, probiotics given after STZ injections [↑](#footnote-ref-9)
