# Supplementary material for: The Effects of Bifidobacterium Probiotic Supplementation on Blood Glucose: A Systematic Review and Meta-Analysis of Animal Models and Clinical Evidence
Source: Adv Nutr. 2023 Nov 2;15(1):100137. doi: 10.1016/j.advnut.2023.10.009 (PMC10831893; doi:10.1016/j.advnut.2023.10.009)
Supplement: Multimedia component 2 [file mmc2.docx]

**Appendix B: Systematic Review and Meta-Analysis of HbA1c in RCTs**

***Systematic Review***

HbA1c was reported in four RCTs, all of which found no differences in HbA1c between parallel placebo and *BF* arms (Ming et al. 2021; Schellekens et al. 2021; Stenman et al. 2016). However, two of the studies reported differences in HbA1c from baseline values. One reported that HbA1c increased in both the placebo and *BF* arms after 12 weeks compared to baseline values (Minami et al. 2015). Specifically, HbA1c increased from 7.0 ± 1.5% at baseline to 7.3 ± 1.5% at 12 weeks in the placebo arm and from 7.5 ± 1.3% to 7.8 ± 1.3% in the *BF* arm; an approximate 3.8% and 4.3% increase, respectively. In this study, most but not all participants were diabetic, and all had elevated BMI and were supplemented with daily capsules of *B. breve* B-3 or placebo for 12 weeks. FBG and glycoalbumin did not change from baseline in either arm. In contrast, Schellekens 2021 found that HbA1c decreased from baseline in the *BF* and placebo arms after 12 weeks of supplementation with daily capsules of *B. longum* APC1472 (Schellekens et al., 2021). Specifically, HbA1c decreased from 5.5 ± 2.5% to 5.2 ± 2.5% in the placebo arm, and from 5.5 ± 2.5% to 5.2 ± 2.4% in the probiotic arm. This represented an approximate 5% decrease in both arms although neither was hyperglycemic at baseline. One study reported no differences in HbA1c in the probiotic arms from baseline (Ming et al., 2021).

***Meta-Analysis***

Four studies including 469 subjects reported HbA1c and were included in a meta-analysis. Since all but one study had normoglycemic baseline HbA1c (< 7%), a subgroup analysis of populations with elevated HbA1c was not possible. One influential study was removed from the effect size estimates, which did not change significance. The mean differences within each study and the pooled MD were low (MD = 0.13% [-0.60, 0.86], t = 0.78, *P* = 0.52, *d* = 0.05 [-0.36, 0.34]), and heterogeneity was correspondingly low (*I^2^* = 0.0% [0.0, 89.6], τ^2^ = 0.0 [0, 3.49]) (**Supplementary Figure 6**). Thus, *BF* supplementation did not affect HbA1c in a mostly normoglycemic population. Publication bias was minimal (**Supplementary Figure 7**).
